# Genetic Diversity in Nitrogen Fertiliser Responses and N Gas Emission in Modern Wheat

**DOI:** 10.3389/fpls.2022.816475

**Published:** 2022-05-04

**Authors:** Maria Oszvald, Kirsty L. Hassall, David Hughes, Adriana Torres-Ballesteros, Ian Clark, Andrew B. Riche, Sigrid Heuer

**Affiliations:** ^1^Plant Science Department, Rothamsted Research, Harpenden, United Kingdom; ^2^Computational and Analytical Sciences, Rothamsted Research, Harpenden, United Kingdom; ^3^Sustainable Agriculture Sciences, Rothamsted Research, Harpenden, United Kingdom; ^4^Department of Crop Improvement and Resilience, NIAB, Cambridge, United Kingdom

**Keywords:** *Triticum aestivum* L, nitrogen fertiliser, N_2_O greenhouse gas, genetic diversity, glutamine synthetase, drought

## Abstract

Crops assimilate nitrogen (N) as ammonium via the glutamine synthetase/glutamate synthase (GS/GOGAT) pathway which is of central importance for N uptake and potentially represents a bottle neck for N fertiliser-use efficiency. The aim of this study was to assess whether genetic diversity for N-assimilation capacity exists in wheat and could be exploited for breeding. Wheat plants rapidly, within 6 h, responded to N application with an increase in GS activity. This was not accompanied by an increase in GS gene transcript abundance and a comparison of GS1 and GS2 protein models revealed a high degree of sequence conservation. N responsiveness amongst ten wheat varieties was assessed by measuring GS enzyme activity, leaf tissue ammonium, and by a leaf-disc assay as a proxy for apoplastic ammonia. Based on these data, a high-GS group showing an overall positive response to N could be distinguished from an inefficient, low-GS group. Subsequent gas emission measurements confirmed plant ammonia emission in response to N application and also revealed emission of N_2_O when N was provided as nitrate, which is in agreement with our current understanding that N_2_O is a by-product of nitrate reduction. Taken together, the data suggest that there is scope for improving N assimilation capacity in wheat and that further investigations into the regulation and role of GS-GOGAT in NH_3_ emission is justified. Likewise, emission of the climate gas N_2_O needs to be reduced, and future research should focus on assessing the nitrate reductase pathway in wheat and explore fertiliser management options.

## Introduction

Nitrogen (N) is essential for plant growth and is a primary driver of crop yield and grain quality. One of the main challenges for modern agriculture is to reduce the use of N-fertiliser, whilst maintaining or increasing grain yield (Hirel et al., 2011; Hawkesford, 2014). Currently, the amount of applied fertiliser required to reach the potential yield of wheat in the United Kingdom, which is estimated at 20 t ha^–1^, ranges from 150 kg to 350 kg ha^–1^ (Mitchell and Sheehy, 2018). However, generally less than half of the applied N-fertiliser is assimilated by crops (Masclaux-Daubresse et al., 2010) and the runoff of excess N contaminates water ways and causes emission of gaseous ammonia (NH_3_) and the potent greenhouse gas nitrous oxide (N_2_O) from soil microbial activities (Linquist et al., 2012; Gao et al., 2019).

Plants have developed sophisticated and highly regulated N-uptake systems. Gene families coding for low- and high-affinity NO_3_^–^ and NH_4_^+^ transporters, respectively, have been identified across different plant species and are located in roots, for primary N acquisition, and other organs for internal distribution and storage (for a review see Li et al., 2017). Once in the plant, NO_3_^–^ is reduced to NH_4_^+^ by nitrate reductase (NR) and nitrite reductase (NiR) (Campbell, 1988) which is then assimilated by the glutamine synthetase-glutamate synthase (GS-GOGAT) cycle, generating glutamine (Balotf et al., 2016). The GS-GOGAT cycle is thus central to the ability of plants to assimilate and use N (e.g., Bernard and Habash, 2009; Thomsen et al., 2014).

Genes in the GS-GOGAT pathway have been studied in some detail, revealing that glutamine synthetase occurs in two major forms, one localised to the cytosol (GS1) and the other localised to the chloroplast (GS2) (García-Gutiérrez et al., 2018). In diploid plants, GS1 is usually encoded by three to five genes, predominantly expressed in the vascular tissues and involved in generating glutamine for intercellular N transport (Brugiére et al., 1999; Tabuchi et al., 2005; Cánovas et al., 2007; Bernard et al., 2008). In contrast, GS2 is generally encoded by a single gene and is located in chloroplasts and mitochondria in green tissues (Tingey et al., 1988; Taira et al., 2004). The chloroplastic GS2 isoform assimilates fertiliser NH_4_^+^ and NH_4_^+^ generated by nitrate reduction and photorespiration in leaves, whereas the cytosolic GS1 isoform is the major form present in roots for primary N assimilation from soil (Lam et al., 1996; Ishiyama et al., 2004). GS1 is also the isoform mainly responsible for N remobilisation during leaf senescence and grain filling (Zhang Z. et al., 2017). In wheat, an initial study had identified ten GS cDNA sequences classified into four subfamilies (GS2, GS1, GSr, and GSe; Bernard et al., 2008). Gene names for the GS1 genes were subsequently updated to GS1;1, GS1;2, and GS1;3 (Wei et al., 2021a) and detailed gene expression and immunolocalisation studies of the proteins showed tissue-specific expression and differential N responsiveness of the individual genes (Wei et al., 2020, 2021b).

A range of plant species have been modified to overexpress cytosolic GS1, following different strategies with regards to the promoter used and transgene selection (for review see Thomsen et al., 2014; Li et al., 2017). The outcomes of these studies have been variable but underscored the importance of cytosolic GS1 for efficient N assimilation, plant growth, and biomass accumulation. For instance, in barley it has been shown that *cis*-genic overexpression of HvGS1-1 improved grain yield and nitrogen use efficiency (NUE), which is encouraging (Gao et al., 2019). In light of the importance of this pathway, there are surprisingly few studies that have investigated whether natural variation in GS enzyme activity exists. Evidence for this has been provided in studies in rice (Kumagai et al., 2011a,b) and tomato (Sánchez-Rodríguez et al., 2011), and a recent study in durum wheat showed differences in GS activity and GS gene expression in genotypes with contrasting grain protein content (Nigro et al., 2016). In the present study, we are complementing these findings with a comparative study of GS enzyme activity and gene expression in response to N fertiliser in ten bread wheat genotypes, providing further evidence that natural variation does exist that can be exploited for breeding.

Whilst NH_4_^+^ is essential for plant growth and development, at high micromolar concentrations it is toxic (Britto and Kronzucker, 2002) and it has been suggested that tolerance is related to N assimilation capacity (Cruz et al., 2006). Ammonium toxicity can lead to the inhibition of root and shoot growth and to leaf chlorosis, and it has been related to ionic imbalances, disturbance of pH gradients across plasma membranes and oxidative stress (Gerendás et al., 1997; Britto and Kronzucker, 2002; Bittsánszky et al., 2015; Esteban et al., 2016; Li et al., 2017). High levels of intracellular NH_4_^+^ can also build up via plant-internal processes, such as lignin biosynthesis, protein and nucleic acid degradation, and photorespiration (Taira et al., 2004; Carvalho et al., 2011; Kumagai et al., 2011b; Bittsánszky et al., 2015; Melino et al., 2018), with the latter two especially relevant under senescence and stress, such as drought or high light. Importantly, it has already been shown that GS activity is compromised under drought stress, for example, and it is therefore important to identify genotypes that maintain GS-GOGAT activity under stress to prevent cellular damage and maintain growth (Sánchez-Rodríguez et al., 2011).

Whilst the importance of emission of N gas from intensive agricultural soils is well known and recently re-gained attention in relation to the evolving field of microbiome studies, it is less widely known that N-gas emission also occurs from plants. Apoplastic NH_4_^+^ and NH_3_ emission was first shown more than 20 years ago in studies in barley (Mattsson and Schjoerring, 1996; Mattsson et al., 1997; Pearson et al., 1998), wheat (Parton et al., 1988a,b; Morgan and Parton, 1989), oilseed rape (Husted and Schjoerring, 1995) and grasses (Heckathorn and DeLucia, 1995; Hanstein and Felle, 1999; Mattsson and Schjoerring, 2002; van Hove et al., 2002; Loubet et al., 2012), and more recently also in rice (Kumagai et al., 2011a). First reports on N_2_O emission from plants came from studies in tobacco (Goshima et al., 1999; Hakata et al., 2003) and wheat (Smart and Bloom, 2001) and this was confirmed in more recent studies in wheat (Baruah et al., 2012), as well as in rice (Gogoi and Baruah, 2012) and grasses (Bowatte et al., 2014). Furthermore, an extensive study conducted by Lenhart et al. (2019) measured N_2_O emission from thirty-two plant species and the significance of this was discussed in relation to climate change.

Taken together, there is sufficient justification for further investigation into diversity of N assimilation capacity in crops and this has gained importance with regards to emission of gasses involved in climate change. In this study we therefore assessed allelic variation in GS genes, GS enzyme activity and N-gas emission within United Kingdom bread wheat to explore, within an already elite germplasm pool, whether genetic diversity exists that can be exploited for improving NUE and reducing N-gas emission. The data presented suggest that there are constitutive differences in N assimilation capacity and GS activity within elite modern wheat. The data also confirm that wheat does emit N gasses in response to ammonia and nitrate fertiliser application, and that N_2_O emission is linked to nitrate.

## Materials and Methods

### Plant Material and Growth Conditions

Ten United Kingdom wheat (*Triticum aestivum* L.) varieties were used in this study, including two spring-type bread wheat varieties (Cadenza and Paragon) and eight winter wheat varieties (Alchemy, Brompton, Claire, Hereward, Rialto, Robigus, Soissons, and XL-19). The winter wheat varieties were chosen because they were used as parents for the development of an “elite MAGIC” population (Mackay et al., 2014) and seeds were provided by NIAB, Cambridge, United Kingdom. Seeds of the spring wheat varieties are available at Rothamsted Research, Harpenden, United Kingdom.

Seeds were surface sterilised with 100% EtOH for 2 min and 5% bleach for 15 min. After 5× washing with sterile water, seeds were germinated on wet filter paper at room temperature (RT) for 7 days before transfer into plastic pots (9 × 9 × 10 cm for the genetic diversity study, 13 × 10 × 11 cm for the gas emission experiment, (see below) containing potting mix composed of 75% medium grade peat, 12% screened sterilised loam, 3% medium grade vermiculite, 10% grit (5 mm screened, lime free), 3.5 kg m^–3^ Osmocote Exact (total N 16%) (Supplier: Scotts UK Professional, Ipswich, Suffolk, United Kingdom) and 0.5 kg m^–3^ PG compound fertiliser (Supplier: Yara UK Ltd., Harvest House, Europarc, Grimsby, North East Lincolnshire). For the N-gas emission the same potting mix was used but without Osmocote (see below). Pots were placed on benches in a controlled environment glasshouse and plants were grown at 22°C/16°C (day/night) with 16 h light. To keep the light intensity above 400 μmol m^–2^ s^–1^, supplementary light was supplied by SON-T lighting as required. Plants were watered daily from the bottom with tap water.

### N-Response Time Course Experiment

To assess temporal GS enzyme activity in response to N-fertiliser application, an initial time course experiment was conducted with the two spring wheat varieties Cadenza and Paragon. Plants were grown as described above in a completely randomised design. At the 2–3 tiller vegetative stage each pot was supplied with NH_4_NO_3_ equivalent to 150 kg N ha^–1^ (0.345 g NH_4_NO_3_) and 250 kg N ha^–1^ (0.575 g NH_4_NO_3_), respectively, dissolved in 50 ml distilled water.

The calculation was based on pot area (cm^2^)/ha * amount of N fertiliser * % of the N content of the fertiliser.

N was applied at 10 a.m. and the youngest fully expanded leaf from six replicate plants for each treatment were harvested at 2, 6, 24, and 30 h after N application and immediately frozen in liquid N and stored at −80°C for further analysis. Water was supplied to control plants which were always sampled in parallel.

### Dry-Down Experiment

To assess the effect of water-stress on N responses, a dry-down pot experiment was carried out with Cadenza and Paragon. Plants were grown as described above and watered daily from the bottom with tap water. At the 3–4 tiller stage watering was discontinued for a subset of the plants to induce water stress. In a pilot study, leaf water content was measured during the dry down period as an indicator of water stress. For this, sampled leaves were weighted (W) before incubation in tap water for 3–4 h in the light at RT. After tapping the leaves dry leaves were weighed (TW) and samples were oven dried 80°C for 24 h and dry weight (DW) determined. Relative water content was calculated as RWC (%) = [(W-DW)/ (TW-DW)] × 100. For subsequent experiments, samples at 72 h after end of watering were collected, when RWC was reduced by about 20% compared to control plants.

### Genetic Diversity Experiment

Eight winter wheat varieties (see above), in addition to Paragon and Cadenza, were grown in a randomised complete block design with six replicates for each treatment in the greenhouse as described above. Plants at the vegetative state (4–5 weeks old plants) were treated with the equivalent of 250 kg N ha^–1^ supplied as NH_4_NO_3_ (0.575 g per pot) and urea (0.264 g per pot), respectively, dissolved in 50 ml de-ionised water. De-ionised water was supplied to control plants. Plants were treated at 10 a.m. and the youngest fully expanded leaf of individual plants was harvested before (0 h), and at 6 and 30 h after N application. Control plants were always sampled in parallel. Samples for the leaf-disc ammonium assay (see below) were taken and directly transferred into buffer. The remainder of the leaf was frozen in liquid N and stored at −80°C for analyses of GS activity and quantification of tissue ammonium (see below).

### Field Experiment

The field site was located at the Rothamsted Experimental Farm (Harpenden, United Kingdom) and utilised the long-term Wheat Genetic Improvement Network (WGIN) Diversity Trial, which was in its 16th year at the time of this experiment (2019). The experiment was set up as a fully randomised split plot design, with three replicates. Plots (1.8 × 9 m) were sown on 09/10/2018 and standard farm protocols were used for crop protection. Three levels of N fertilisation were applied in three splits, amounting to a total seasonal N doses equivalent to 100 (N1), 200 (N2), and 350 (N3) kg N ha^–1^, respectively. For the present study, two selected varieties (Cadenza and Paragon) were sampled from the three replicate N1 and N3 plots, directly before (T0) and at 48 h after application of the second N split (50 kg N ha^–1^ for N1; 250 kg N ha^–1^ for N3). Both plots had previously received 50 kg N in the first split application (February) and received a final, third split application of 50 kg N in May. Leaf samples of six randomly selected plants from each plot were immediately frozen in liquid N and stored at −80°C for further analysis.

### Measuring N-Gas Emission Using a Picarro Gas Analyser

Three selected genotypes (Cadenza, Paragon, and Claire) were grown under controlled conditions as describe above in a randomised complete block design to measure N gas emission using a Picarro G2508 Greenhouse Gas Analyser (Picarro Inc., Santa Clara, CA, United States). One-week-old seedlings were transplanted into pots (13 × 10 × 11 cm) with potting mix (see above) without additional nutrients. After 24 h, plants were supplied with a modified Letcombe’s solution according to Masters-Clark et al. (2020). For the low-N treatment, 0.325 ml of KNO_3_ (1.6 M) was supplied to each pot and 3.25 ml were supplied for the high-N treatment. Each pot received the same amount of P (3.25 ml, 0.17 M KH_2_PO_4_) and micronutrients (Masters-Clark et al., 2020). Plants were kept well-watered using tap water. For the gas measurement, plants at the 3–4 tiller stage were treated with the equivalent of 250 kg ha^–1^ N supplied either as KNO_3_ or NH_4_NO_3_. Water was supplied to controls. The soil surface of each pot was covered with aluminium foil and cling film before the Picarro clear plexiglass chambers were mounted and sealed with tape. As a soil control, pots were used from which the above-ground plant biomass was removed to account for any emission coming from roots and/or the soil microbiome. These pots were treated the same way as pots with plants. The experiment was carried out in six replicates for each genotype and treatment. The Picarro instrument was set up to measures sixteen chambers for 3:49 min each consecutively and repeatedly over 24 h. Data output from the G2508 was on average every 2 s as dry mole fraction in ppb for NH_3_ gas, and ppm for N_2_O. The flow rate between the G2508 and incubation vessel and chamber were 250 mL min^–1^ using a low-leak external vacuum pump. At the end of the experiment, above-ground biomass was oven dried at 80°C for 24 h to determine the dry weight, which was used to normalize maximum NH_3_ and N_2_O gas emissions.

A second experiment was carried out to compare Cadenza and Paragon with an elite wheat from a comparably low-N environment and for this the variety Mace (AGT, Australia) was used. The experiment was conducted as described above except that plants were grown under high-N conditions in potting mix supplemented with Osmocote (see above). Plants were treated with the equivalent of 250 kg N ha^–1^ supplied as NH_4_NO_3_. Water was supplied to controls.

A third experiment was carried out to investigate if N_2_O gas emission is derived from nitrate reduction and/or ammonia assimilation. For this, Cadenza plants were grown under low N conditions as described above and treated with the equivalent of 250 kg N ha^–1^ supplied as urea and KNO_3_ respectively with and without 150 μM of the nitrate reductase inhibitor (NI) tungsten sulphate. Water was supplied to controls.

### Glutamine Synthetase Enzyme Assay

Total (combined GS1 and GS2) extractable GS activity was determined in 100 mg ground fresh leaf material extracted in a 1 mL buffer solution containing 25 mM TRIS–HCl, 1 mM EDTA, 1 mM DTT, 1 mM MgCl_2_, 0.2 g polyvenyl pyrrolidone and 2 mM leupeptin, pH 7.6. GS activity was measured according to O’Neal and Joy (1974) by incubating 50 μl leaf extracts in an assay medium containing 600 mM glutamate, 45 mM hydroxylamine hydrochloride, 150 mM MgSO_4_*7H_2_O, 30 mM EDTA-Na2, and 60 mM ATP for 30 min at 30°C in a shaker incubator. The enzyme reaction was terminated with 300 μl stopping solution (0.67M FeCl_3_, 0.2M TCA, 0.7M HCl). After centrifugation (5 min, 13,000 × *g*) the optical density of supernatants was measured at 540 nm using a Jenway 6715 UV/Vis spectrophotometer.

### Leaf-Disc Ammonium Measurements

For the present study, a protocol for the extraction of apoplastic ammonium (Husted and Schjoerring, 1995) was modified and we refer to this method as “leaf-disc” ammonium assay.

For this, four leaf-discs (5 mm diameter) from the middle of a fully expanded leaf were cut with a hole punch and immediately submerged in 1 ml isotonic sorbitol solution (0.28M) in an Eppendorf tube. Samples were incubated at 4°C for 24 h before vacuum infiltration in a desiccator (DURAN, DN 150) for 2 min at a pressure of 4 atm. In contrast to the published protocol, leaf-discs were centrifuged within the sorbitol solution (2,000 × *g*, 5 min, 4°C) and aliquots (10 μl) of the supernatant were used to measure NH_4_^+^ concentration using the Megazyme rapid Ammonia kit (K-AMIAR 04/18). This assay is based on reductive amination of 2-oxoglutarate with glutamate dehydrogenase (GDH) and NADPH. Assays were always performed with two technical replicates and ammonium concentration was normalised to leaf-disc weight.

The absence of cytosolic contamination of the leaf-disc extract with cytoplasm was confirmed by measuring GS enzyme activity (see below) and activity of the cytosolic glycolysis enzyme G6PDH according to Lohr and Waller (1974). Briefly, G6PDH activity is measured in a spectrophotometer following the reduction of NADP in a solution containing 66 mM KH_2_PO_4_/K_2_HPO_4_ (pH 7.6), 10 mM MgCl_2_, 300 mM NADP, 2 mM glucose-6-phosphate (G6P) and 10 μl of leaf tissue extracts in a final volume of 300 μl. G6PDH activity in the samples was calculated using a standard curve for NADPH at 340 nm (Spectra 340PC plate reader). All assays were performed in two technical replicates.

### Quantification of Ammonium, Nitrate, Glutamine and Total Protein in Leaf Tissue

To determine the NH_4_^+^ concentration in leaf tissue, 100 mg of fully expanded leaf samples were ground in liquid nitrogen and incubated in 1 ml 0.05M H_2_SO_4_ at RT for 1 h. The homogenate was centrifuged at 13,000 × *g* for 15 min at RT and the supernatant was used to quantify NH_4_^+^ using the Megazyme rapid ammonia kit (K-AMIAR 04/18) according to the manufacturer’s instructions.

The nitrate content in leaves was determined in water extracts of 100 mg ground leaf tissue according to Zhao and Wang (2017). Samples were mixed with 100 μl of deionised water and boiled at 100°C for at least 20 min. Deionised water (0.1 ml) was used as a control. After centrifugation at 15,871 × *g* for 10 min, the supernatant was transferred into a new tube and 40 μl salicylic acid-sulphuric acid was added. The reaction was incubated at RT for 20 min before 0.95 ml of 8% (w/v) NaOH solution was added and tubes were allowed to cool to RT for 20–30 min. The OD_410_ value of each sample was measured in a spectrophotometer and water was used as a blank. The nitrate concentration was calculated as Y = CV/W [Y: nitrate content (μg/g); C: nitrate concentration calculated with OD_410_ using a regression equation from the standard curve (μg/ml); V: total volume of extracted sample (ml); W: weight of sample (g)].

Glutamine was extracted from 50 mg frozen, ground leaf samples with 0.5 ml 80% EtOH for 3 h at 40°C in a heating block and centrifuged at 10,000 × *g* for 15 min at RT. The supernatant (10 μl) was used to determine leaf L-glutamine content using the L-glutamine/ammonia rapid kit from Megazyme (K-GLNAM 04/17). Samples were normalised to 100 mg FW.

The total soluble protein concentration in leaves was measured using the Bradford method with BSA as standard (Bradford, 1976).

### Glutamine Synthetase Genes in Five Wheat Genomes

Genomic sequences of the wheat *GS1* and *GS2* genes were initially retrieved from the Chinese Spring reference genome (IWGSC, INSDC Assembly GCA_900519105.1; July 2018) available in Ensembl Plants.^1^ Accession numbers are provided in Table 1. Representatives of the different GS1 and GS2 homeologs were then used for a BLASTp search in Ensembl Plants to ensure all *GS* genes and homeologs had been identified. This search revealed an additional low confidence (LC) *GS1* homeolog on chromosome 6D. The chromosomal position of the individual sequences was derived from Ensembl Plants. The genomic sequences of representative Chinese Spring *GS1* and *GS2* homeologs were then used for a BLASTn search of the available genomic scaffolds of four wheat varieties (Cadenza, Paragon, Robigus, and Claire)^2^ and a tetraploid durum wheat (Kronos) (sequence versions 2018-02-19).^3^ The identified GS-gene containing scaffolds (Supplementary Table 1) were aligned to the genomic sequence of the respective GS Chinese Spring gene sequences using the LASTZ algorithm of the Geneious 10.2.3 software and the aligned regions with some 5′ and 3′ flanking regions were extracted. Gene models were predicted by aligning the extracted scaffold sequences with the respective Chinese Spring genomic sequence, the longest cDNA, and the corresponding coding regions (CDR) retrieved from Ensembl Plants. Extraction of the putative CDRs, translation into protein, and sequence alignments using the MAFFT algorithm were conducted using Geneious 10.2.3. A comparison of the predicted protein sequences to create a circular tree was done in Geneious. Additional gene expression data were derived from the wheat expression database.^4^

**TABLE 1:** Putative glutamine synthetase (GS1 and GS2) genes from the Chinese Spring reference genome.

Chromosome	Ensembl Plant accession numbers	Gene name Bernard et al., 2008	Gene name Wei et al., 2021a	Homeolog gene name
Chr 2A	TraesCS2A02G500400	GS2a	TaGS2	*TaGS2-2A*
Chr 2B	TraesCS2B02G528300	GS2b		*TaGS2-2B*
Chr 2D	TraesCS2D02G500600	GS2c		*TaGS2-2D*
Chr 4A	TraesCS4A02G063800	None	TaGS1;2	*TaGS1;2-4A*
Chr 4B	TraesCS4B02G240900	GSr1		*TaGS1;2-4B*
Chr 4D	TraesCS4D02G240700	GSr2		*TaGS1;2-4D*
Chr 4A	TraesCS4A02G266900	None	TaGS1;3	*TaGS1;3-4A*
Chr 4B	TraesCS4B02G047400	GSe1		*TaGS1;3-4B*
Chr 4D	TraesCS4D02G047400	GSe2		*TaGS1;3-4D*
Chr 6A	TraesCS6A02G298100	GS1a	TaGS1;1	*TaGS1;1-6A*
Chr 6B	TraesCS6B02G327500	GS1b		*TaGS1;1-6B*
Chr 6D	TraesCS6D02G383600LC	Gs1c		*TaGS1;1-6D*

### Quantitative RT-PCR

Total RNA was extracted from 50 mg frozen, ground leaf samples using TRIzol reagent (Invitrogen Cat No. 15596-026) (Chomczynski and Sacchi, 1987). Integrity of the RNA was confirmed in a 2% (w/v) agarose gel and RNA concentration was quantified using an ND-1000 spectrophotometer (NanoDrop Technologies, United States). cDNA synthesis was performed with 2 μg of total RNA per sample using Invitrogen Superscript III reverse transcriptase with dT-adapter primers (Invitrogen, United Kingdom) in a 20 μl reaction according to the manufacturer’s protocol. cDNA quality was verified by PCR amplification of the actin gene (for primer sequences see Table 2).

**TABLE 2:** Sequences of primers used for qPCR analysis.

	Gene	Forward primer (5′-3′)	Reverse primer (5′-3′)	Size (bp)	Tm (°C)
Target genes	*TaGS1*	ATCAACACCTTCAGCTGGGG	GAAGTAGCCCTTGCCGTTCT	87	60
	*TaGS2*	GAAATCAGTGGAACAAACGG	CTCCCGCATCAATACCAAC	82	60
Reference genes	*Ta Actin*	GACAATGGAACCGGAATGGTC	GTGTGATGCCAGATTTTCTCCAT	236	60
	*Ta 2526.1.S1_at*	CGAGATCGACCAAGAATGG	TGAGTGTTGCCTCCCTCC	227	60
	*Ta GAPdH*	TTCAACATCATTCCAAGCAGCA	CGTAACCCAAAATGCCCTTG	197	60

For the quantification of gene transcripts, the SYBR^®^ Green Jump Start*™* Taq Ready Mix*™* (Sigma-Aldrich, United Kingdom) was used in the Applied Biosystems 7500 Real Time PCR System. Each 25 μl reaction contained 0.5 μl cDNA and 10 μM forward and reverse primers, respectively.

For primer design, detailed comparative nucleotide sequence analyses of the above-described sequences were conducted to identify gene- and homeolog-specific regions and primer pairs were positioned manually in selected regions (data not shown). For the purpose of this study, primers were designed targeting regions highly conserved within GS2 and the GS1 genes, respectively, specifically annealing to and amplifying the three *GS2* homeologs together, as well as the three *GS1* genes and their corresponding homeologs combined. Efficiency of at least three primer pairs were tested in cDNA dilution series and the mean primer efficiency was estimated using the linear phase of all individual reaction amplification curves (Ramakers et al., 2003), calculated by using the LinRegPCR package (Tuomi et al., 2010). Three house-keeping reference genes (Table 2) were used to normalise relative quantification (NRQ) of expression (NE). The NRQ of NE was calculated in relation to the CT values, the primer efficiency (E) of the target gene (X), and the normalising reference gene (N) as Normalised Relative Expression NRE using the formula: NRE = (EX)-CT,X/(EN)CT,N according to Rieu and Powers (2009).

### Statistical Analysis

Statistical significance was assessed using ANOVA and Student’s *t*-test in Genstat 20th Edition (VSNi) and in Microsoft Excel. For both, the drought and genetic diversity experiments no blocking terms were included. For the field trial the block structure block/whole plot/plot was used to reflect the split-plot structure of N application to whole plots. The genetic diversity and field trials consisted of the three-way factorial treatment structure genotype×treatment×time. The drought experiment consisted of the treatment structure [baseline/(time×treatment)]×genotype, where baseline is indicative of the first time point sampled before any treatments were applied. Where appropriate, response variables were transformed to ensure homogeneity of variance, specifically concentration of leaf-disc ammonium was analysed on the square root scale and tissue ammonium was analysed on the scale of the natural logarithm. Small levels of missingness in these three variables were present across the three experiments and were estimated iteratively through the ANOVA. Student’s *t*-test was applied to test the significance of differences between the data from Cadenza and Paragon in the time course experiment and in the gene expression analysis. For the leaf NO_3_^–^ and glutamine measurements, a regression analysis was performed, and the *F*-statistics reported are the sequential *F*-statistics associated with adding terms in the order time, treatment, genotype and associated higher order interactions. Different orders of fitting were tested and although quantitative results deviated marginally, conclusions remained qualitatively the same. For the comparative analysis of the N response in ten genotypes, mean values were based on sqrt (raw data leaf-disc ammonia) and log2 transformation of data [LFoldChange = log2(treatment/control Mean)]. For the Picarro experiment, ANOVA was used to compare emissions from plant vs. soil using the raw data. To compare emissions between genotypes and treatment effects, ANOVA was carried out on the normalised data with treatment structure comprised of: Genotype*(Trt_type/Treatment)*N.level. To ensure homogeneity of variance, all response variables of the maximum emission data were first log transformed.

## Results

### Temporal Response of Glutamine Synthetase to N Application

**FIGURE 1:**
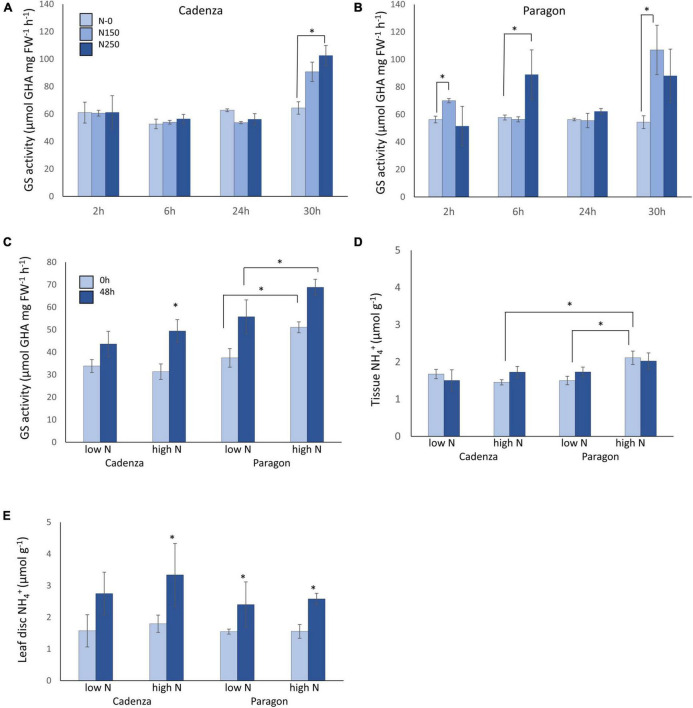
Temporal response of wheat glutamine synthetase to N fertiliser under controlled and field conditions. The response of glutamine synthetase (GS) enzyme activity was measured in two bread wheat genotypes grown in fully fertilised (Osmocote slow-release fertiliser) potting mix. GS activity in leaf samples of Cadenza **(A)** and Paragon **(B)** was measured at four timepoints after application of NH_4_NO_3_, equivalent to 150 kg N ha^–1^ (N150) and 250 kg ha^–1^ (N250), respectively. Water was applied to control plants (N-0) and measured in parallel. Each value represents the mean ± SE of four biological replicates. The same genotypes were sampled from a long-term field experiment with plots adjusted to low (100 kg N ha^–1^) and high (350 kg N ha^–1^) total seasonal N application (see text for details). GS activity **(C)**, tissue ammonium **(D)** and leaf-disc assay ammonium **(E)** were measured in leaf samples collected before (0 h) and at 48 h after the second split application of NH_4_SO_4_ fertiliser equivalent to 50 kg N ha^–1^ (low N plot) and 250 kg N ha^–1^ (high-N plot), respectively. Each value represents the mean ± SE of six biological replicates. Asterisks indicate significant differences between means (*p* < 0.05).

To assess the response of glutamine synthetase (GS) to high N application a time-course experiment was conducted using the United Kingdom spring wheat varieties Cadenza and Paragon at the vegetative stage grown in fully fertilised potting mix (Figures 1A,B). Significant differences in GS enzyme activity were observed over time (*F*_3,66_ = 16.65, *p* < 0.001), with this temporal trend varying both with N treatment (*F*_6,66_ = 2.72, *p* = 0.02) and with genotype (*F*_3,66_ = 3.13, *p* = 0.031) Under control conditions (zero additional N), GS activity was similar in both genotypes at all time points with a GS activity around 60 μmol GHA mg FW^–1^ h^–1^ NH_4_^+^ (Figures 1A,B). The application of the equivalent of 150 kg N ha^–1^ (as NH_4_NO_3_) significantly increased GS activity at 2 h and at 30 h in Paragon (Figure 1A) whereas there was no significant GS response in Cadenza to this N level (Figure 1B). Application of the higher N250 doses had a significant positive effect on GS activity in Paragon at 6 h and in Cadenza at 30 h, increasing GS activity to about 90 and 100 μmol GHA mg FW^–1^ h^–1^, respectively.

### Plant Responses to N-Fertiliser Application in the Field

To validate the results from the pot experiment, the response to different levels of N-fertiliser application was analysed in vegetative stage Cadenza and Paragon plants grown in a long-term field experiment with low and high N-plots (see section “Materials and Methods” for details). To assess N responses, plants were sampled before and at 48 h after N application and analysed for GS enzyme activity and tissue NH_4_^+^, as well as for leaf-disc NH_4_^+^ (Figures 1C–E). A significant change in GS activity was seen over time (*F*_1,117_ = 43.62, *p* < 0.001). In Cadenza, GS activity prior to N application was similar in the low-N and the high-N plots and a significant increase in GS activity after fertiliser application was observed only in plants grown in the high-N plot (Figure 1C). In contrast, GS activity in Paragon before N application was already significantly higher in the high-N plots and significantly increased further after N application in both, the low-N and high-N plots (up to 68.81 μmol GHA g FW^–1^ h^–1^; Figure 1C).

In contrast to the observed changes in GS enzyme activity, tissue NH_4_^+^ concentration did not respond to N-fertiliser application (*F*_1,115_ = 2.39, *p* = 0.125). However, whilst, tissue NH_4_^+^ was similar (between 1.49 and 1.72 μmol g^–1^) in both genotypes under low-N conditions, Paragon had a significantly higher tissue ammonium concentration under high N conditions (2.11 μmol g^–1^ FW) compared to plants grown in the low N plots (1.49 μmol g^–1^) (Figure 1D). Interestingly, leaf-disc extracted ammonium, used here as a proxy for potential volatile NH_3_ losses, increased significantly (*F*_1,128_ = 31.85, *p* < 0.001) in response to N application in the high N plot in Cadenza (from 1.82 to 3.10 μmol g^–1^) and in both, the high and low N plots in Paragon, although it remained comparably lower in the latter (max 2.73 μmol g^–1^) (Figure 1E).

### Allelic Variation and Expression of Glutamine Synthetase Genes in Wheat

**FIGURE 2:**
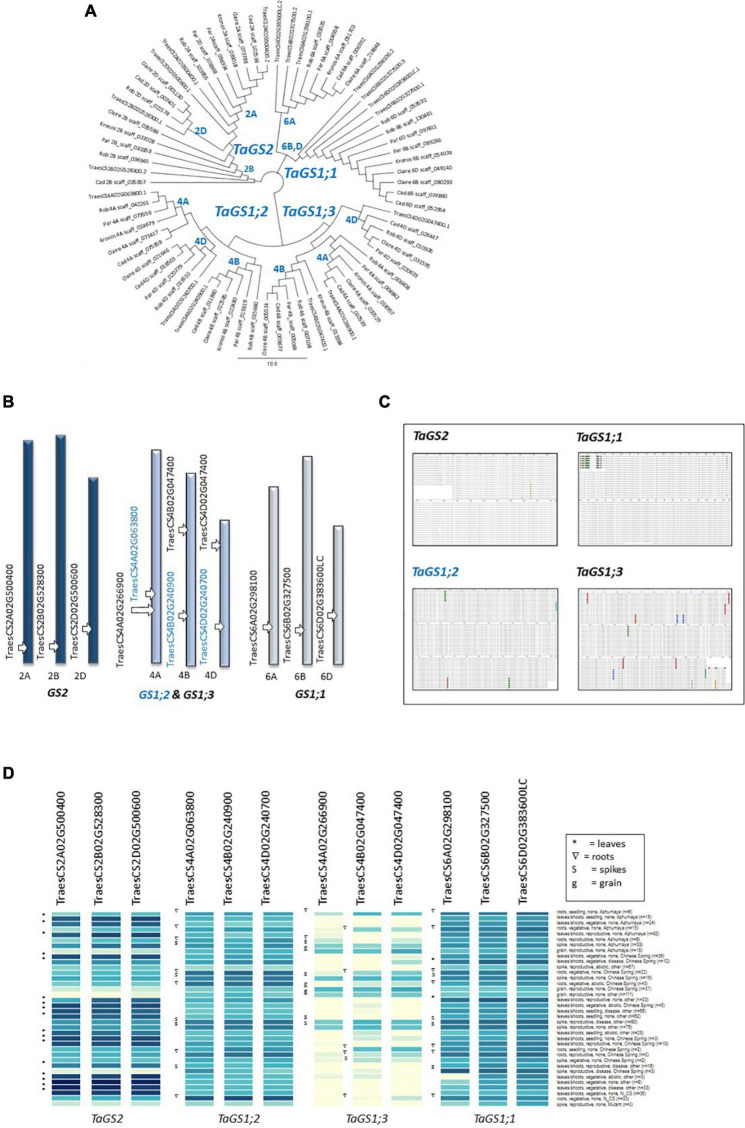
Comparison of glutamine synthetase genes in six wheat varieties. Sequences of the wheat GS1 and GS2 genes of the reference Chinese Spring reference genome were extracted from Ensembl Plants (https://plants.ensembl.org/Triticum_aestivum) and used as a query and template to extract genomic sequences from publicly available scaffolds and to predict GS1 and GS2 protein models in four bread wheat varieties (Cadenza, Paragon, Claire, and Robigus) and one durum wheat (Kronos) (see text for details; Supplementary Table 1). A comparison of the protein models is shown in the tree generated in Geneious **(A)**. The gene IDs and chromosomal positions of the genes in the Chinese Spring reference genome are shown in **(B)**. The high degree of conservation of the genes across the six analysed varieties is shown in the alignment in **(C)** (see Supplementary Figures 1–3 for details). Available gene expression data (http://www.wheatexpression.com) show a preferential expression of GS2 in leaves, and the additional expression of GS1 genes in roots, spikes and grain **(D)**. Note that the GS1;3 genes show a distinct expression pattern and had the highest number of amino acid changes **(C)** (http://www.wheatexpression.com; Ramírez-González and Borrill, 2018).

Glutamine synthetases constitute a multi-gene family in wheat (Figure 2A) and for the purpose of this study the sequence information has been updated based on recent versions of the Chinese Spring reference genome (IWGSC RefSeq v1.0; International Wheat Genome Sequencing Consortium (IWGSC) et al., 2018) and complemented with protein models based on available genomic scaffold sequences of five wheat varieties (Figure 2 and Supplementary Table 1). In Chinese Spring, the *GS2* gene is located on chromosome group 2, two GS1 genes (*GS1;2* and *GS1;3*) are located on chromosome group 4, and a third *GS1* gene (*GS1;1*) is located on chromosome group 6 (Figure 2B).

Using the Chinese Spring genomic sequences as a query, *GS1* and *GS2* genomic sequences available via Decypher were identified in four bread wheat varieties (Cadenza, Paragon, Robigus, and Claire), as well as in the tetraploid durum wheat variety Kronos. For the prediction of the protein models, Chinese Spring genomic intron-exon structures were then superimposed onto the assembled genomic sequences and exons were extracted and translated into putative protein sequences using Geneious (see section “Materials and Methods” for details). Details on the wheat scaffolds from which sequences were retrieved and gene names are provided in Table 1 and Supplementary Table 1.

A comparison of the predicted protein models across the six analysed wheat varieties revealed a very high degree of conservation with 98–100% sequence identity over the entire length of the protein (Figure 2C and see Supplementary Figures 1–3 for full size alignments). The highest degree of sequence variation was observed for the *TaGS1;3* gene on chromosome 4 with thirteen amino acid substitutions (Figures 2A,C). Based on available expression data^5^ GS2 is predominantly expressed in leaves (Figure 2D) in agreement with its localisation in the chloroplast and role in photorespiratory N re-assimilation. The cytoplasmic GS1 genes are predominantly expressed in roots and spikes/grain consistent with their role in primary N assimilation in the roots and N remobilisation during senescence and grain filling (Figure 2D). Whereas the *TaGS1;1* homeologs on chromosome 6 and *TaGS1;2* homeologs on chromosome 4 are also expressed in leaves, the more variable chromosome 4 *TaGS1;3* gene appears not to be expressed in leaves (Figure 2D).

**FIGURE 3:**
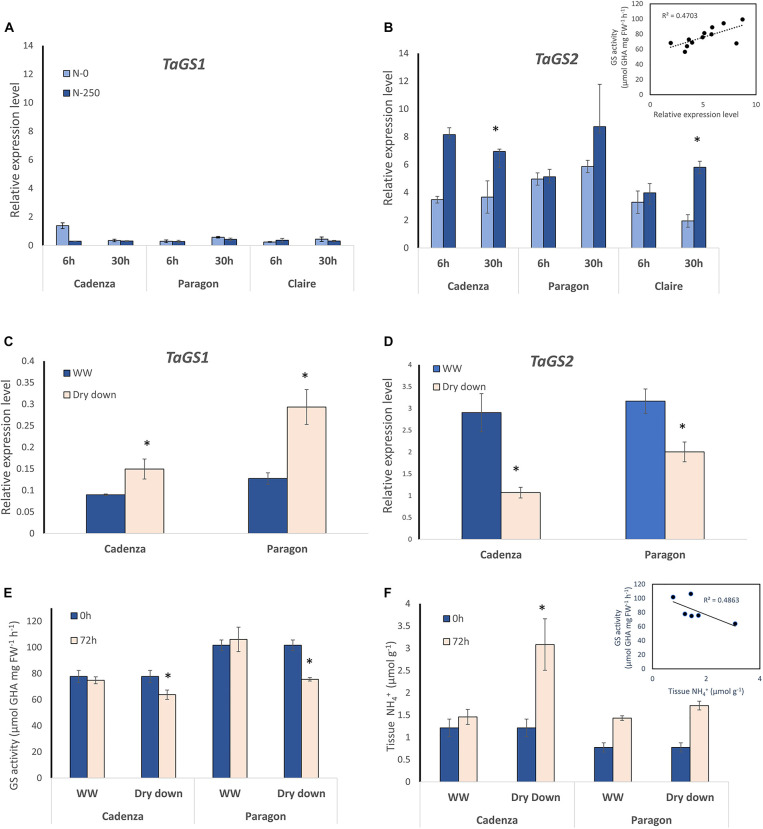
GS1 and GS2 gene expression analysis in response to N and water deficit. For gene expression analyses, specific primers were designed that specifically amplify the GS1 genes and GS2 genes, respectively (see Figure 2). Expression of the three GS1 genes and their corresponding homeologs **(A)** and the GS2 gene with its three homeologs **(B)** was quantified in leaf samples from the four indicated wheat genotypes at 6 h and 30 after NH_4_NO_3_ application equivalent of 250 kg N ha^–1^ (N250) and in water-treated control plants (N-0). Each value represents the mean ± SE of four biological replicates. The inlay in **(B)** shows the correlation of GS gene expression and GS enzyme activity (corresponding GS activity data are shown in Supplementary Figure 4). The response of GS1 **(C)** and GS2 **(D)** gene expression to a dry-down treatment was analysed at 72 h after withholding water. Well-watered (WW) plants were sampled in parallel. Each value represents the mean ± SE of four biological replicates. In the dry-down experiment, GS enzyme activity **(E)** and leaf tissue ammonium content **(F)** were measured in parallel at 0 h and at 72 h after withholding water. The inlay shows a negative correlation of tissue ammonium and GS activity. Each value represents the mean ± SE of six biological replicates. Asterisks indicate significant differences between means (*p* < 0.05) of each treatment.

To enable gene expression analysis, detailed DNA sequence comparisons between the six genotypes were conducted and, for the purpose of this study, primers were designed that specifically bind to the three GS2 homeologs, as well as GS1-specific primers that bind to all homeologs of the three GS1 genes (for primer sequences see Table 2). Based on the respond in the GS activity in the different genotypes, N-responsiveness of the GS genes was assessed in three selected genotypes (Cadenza, Paragon, Claire), grown under N replete conditions, at the vegetative stage before and at 6 and 30 h after N application (NH_4_NO_3_; equivalent of 250 kg N ha^–1^). In the analysed leaf samples, combined expression of the *GS1* genes was much lower than expression of the *GS2* homeologs and showed no significant N response (Figures 3A,B). Contrary to our expectation, increases in *GS2* gene expression after N application were also largely not significant (Figure 3B) although there was a weak (*r* = 0.4459) correlation between GS activity and *GS2* gene expression (see inlay in Figure 3B).

In order to corroborate lack of transcriptional regulation of GS genes, gene expression in Cadenza and Paragon was assessed in response to water stress. As had been overserved in the previous experiment, under water stress, *GS1* transcript abundance was about 10-times lower compared to *GS2* in both genotypes, however, *GS1* transcript abundance significantly increased in response to water stress (*F*_11_ = 7.921, *p* < 0.02) whilst *GS2* transcript abundance was significantly reduced (*F*_11_ = 17.660, *p* < 0.001) (Figures 3C,D). The parallel analysis of GS enzyme activity showed a significant difference between genotypes (*F*_1,67_ = 9.13 *p* = 0.004) with Paragon having higher GS activity on average (92.8 μmol GHA g FW^–1^ h^–1^) than Cadenza (76.9 μmol GHA g FW^–1^ h^–1^) (Figure 3E). In response to the dry-down treatment, GS enzyme activity significantly decreased in both genotypes compared to the well-watered controls by the end of the experiment, at 72 h after withholding water (Figure 3F). In agreement with that, leaf tissue NH_4_^+^ and GS activity were negatively correlated (*r* = 0.486) and increased under water stress (see inlay in Figure 3F).

### Genetic Diversity in Wheat for Plant N-Fertiliser Responses

**FIGURE 4:**
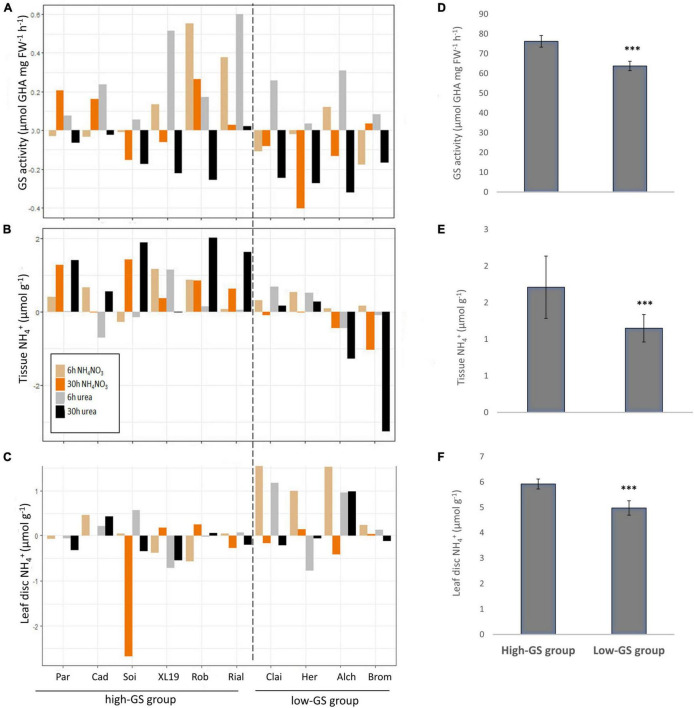
Differential response of ten wheat genotypes to N fertilisers. Ten elite United Kingdom wheat genotypes were treated with the equivalent of 250 kg N ha^–1^ applied either as NH_4_NO_3_ or urea and the response of GS activity **(A)**, leaf tissue ammonium **(B)** and leaf-disc ammonium **(C)** were measured at 6 and 30 h after N application. The data shown represent log2 transformed relative changes compared to water-treated control plants (average values of six replicates). Data were calculated as LFold_Change = log2(treatment/control_mean). Average values of the actual data for the high-GS and the low-GS group, respectively, are given in **(D–F)**. Error bars represent standard errors. Differences between groups were significant at *p* < 0.001. Cad, Cadenza; Par, Paragon; Soi, Soissons; Bro, Brompton; Alc, Alchemy; Her, Hereward; Cl, Claire; XL-19, XL-19; Rob, Robigus; Ria, Rialto. Differences between groups were significant at ***p < 0.001.

Based on the observed positive response of GS activity and increase in leaf-disc NH_4_^+^ in the two analysed spring wheat genotypes Cadenza and Paragon, this study was extended to winter wheat. As mentioned above, the target environment for this study is the United Kingdom intensive system and we therefore chose eight founders of an elite MAGIC population (Alchemy, Brompton, Claire, Hereward, Rialto, Robigus, Soissons, and Xl-19; NIAB Elite MAGIC, Mackay et al., 2014). Plants were grown under N-replete conditions and at the 3–4 tiller vegetative stage were supplied with the equivalent of 250 kg N ha^–1^, either as solubilised NH_4_NO_3_ or as urea and GS enzyme activity, leaf tissue and leaf-disc NH_4_^+^were measured at 6 and 30 h after N application. Control plants were supplied with water and analysed in parallel (Figure 4 and Supplementary Table 2). NH_4_NO_3_ was included in this study to assess if N assimilation might be higher when plants employ both, the plant inherent ammonium and nitrate uptake systems. However, no significant differences between N sources were detected under the experimental conditions applied in this study. In contrast. a strong significant effect of time was seen in GS activity (*F*_1,296_ = 34.46, *p* < 0.001) with this effect differing between treatments (*F*_2,296_ = 10.59, *p* < 0.001). Both leaf-disc and leaf tissue NH_4_^+^ concentration differed significantly across times, treatment and genotype with significant three-way interactions (*F*_18,295_ = 1.80, *p* = 0.024 and *F*_18,288_ = 4.11, *p* < 0.001).

Overall, the data were variable and difficult to interpret, however, two significantly distinct groups could be distinguished, both in absolute GS activity (*F*_1,296_ = 52.85; *p* < 0.001) and in the relative change in GS activity [raw data/*F*_1,296_ = 11,22 (log2 data); *p* < 0.001] based on an ANOVA of log2 transformed change data and actual data (Figures 4A,D and Supplementary Table 2). This differentiation is mainly based on the higher GS activity present in the “high-GS group” (mean 76.2 μmol GHA h^–1^ FW^–1^) compared to the “low-GS group” (mean 63.8 μmol GHA h^–1^ FW^–1^) (Figure 4D). Likewise, the log2-fold change in response to N application was significantly different between the high-GS group (mean 0.09375 log2 fold change) and the low-GS group (mean −0.08875 log2 fold change) (Figure 4A). This grouping was also supported by the tissue NH_4_^+^ concentration [*F*_1,296_ = 13.01 (raw data)/*F*_1,296_ = 45.29 (log 2 data); *p* < 0.001] and leaf-disc NH_4_^+^ data [*F*_1,296_ = 12.97 (sqrt raw data)/*F*_1,296_ = 14.9 (log 2 data); *p* < 0.001], which were both significantly higher in the high-GS group (Figures 4B,C,E,F).

**FIGURE 5:**
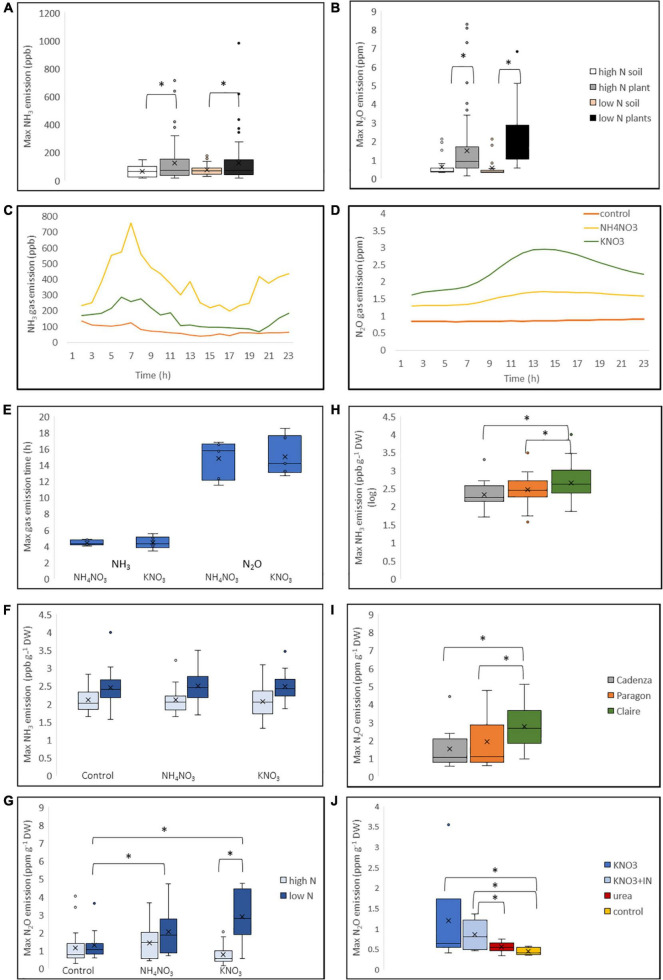
Ammonia and N_2_O gas emission in response to N application in wheat. Three wheat varieties representative of the high-GS group (Cadenza, Paragon) and low GS-group (Claire) were grown under low and high N conditions, respectively, and treated with the equivalent of 250 kg N ha^–1^ applied either as NH_4_NO_3_ or KNO_3_ immediately before gas measurements. Water was applied to control plants measured in parallel. For the soil control, above-ground plant tissue was cut off, but roots were retained (see text for details). Gas emission was measured using a gas analyser (Picarro G2508; United States) over a 24 h period. Maximum NH_3_ **(A)** and N_2_O **(B)** emission from plants was significantly higher than the background emission from soil. Representative processed Picarro data for NH_3_ **(C)** and N_2_O **(D)** emission are shown, with maximum NH_3_ emission occurring earlier than maximum N_2_O emission **(E)**. The effect of the growth conditions and N treatment on maximum NH_3_ and N_2_O emission was calculated using the combined data of the three genotypes showing higher emission from N-starved plants **(F,G)**. Genotypic differences between plants grown under low-N conditions are shown in **(H,I)**. Cadenza plants grown under low-N conditions were treated with urea and KNO_3_, with and with nitrate reductase inhibitor (NI), showing highest N_2_O emission in the KNO_3_ treatment **(J)**. Data are derived from six replicates for each genotype and treatment. Asterisks indicate significant differences between means (*p* < 0.05) of each treatment.

The pattern that emerged from the analysis of this complex data set revealed an overall positive N response of the high-GS group, i.e., generally the six genotypes within that group (Paragon, Cadenza, Soissons, XL-19, Robigus, and Rialto) had a constitutively higher GS activity which tended to respond positively to N application (Figures 4A,D). In contrast, the data suggest a possible inhibitory effect of N application on GS activity in the low-GS group (Claire, Hereward, Alchemy, Brompton; Figures 4A,D). Likewise, tissue NH_4_^+^ concentration in the high-GS group increased after N application and was constitutively higher (mean 1.1 μmol g^–1^ NH_4_^+^) compared to the low-GS group (mean 0.89 μmol g^–1^ NH_4_^+^) (Figures 5B,E). Contrary to this, leaf-disc NH_4_^+^, as a proxy for potential N losses via volatile NH_3_ gas, increased in the low-GS group (Figures 4C,F). However, although in the high-GS group leaf-disc NH_4_^+^ did not increase in response to N, it was overall higher in this group (mean 5.5 μmol g^–1^ NH_4_^+^) compared to the low GS-group (4.5 μmol g^–1^ NH_4_^+^) (Figure 4F).

To further investigate the observed genotypic differences, three genotypes were selected to additionally determine changes in leaf nitrate and L-glutamine content in response to NH_4_NO_3_ application. Claire was selected as a representative of the low-GS group and Cadenza and Paragon as representatives of the high-GS group. The combined data of three selected genotypes, showed a significant increase in leaf tissue L-glutamine, NO_3_^–^ and NH_4_^+^ content, as well as in GS activity, in response to N application (Supplementary Figures 4A–D). Of the three genotypes, Claire had the lowest GS activity and leaf-disc NH_4_^+^, as well as the lowest L-glutamine and tissue NH_4_^+^, although the latter differences were not significant (Supplementary Figures 4F–J). Overall, there was a positive correlation of GS activity with leaf L-glutamine content (*r* = 0.58), as well as tissue NH_4_^+^ and leaf-disc NH_4_^+^ (*r* = 0.54; *r* = 0.56). Total soluble protein content of samples was also analysed but did not show any changes in response to N application at the two timepoints (6 and 30 h) analysed (data not shown).

### N Gas Emission From Plants Grown Under Low and High N Conditions

To corroborate the leaf-disc NH_4_^+^ data and confirm that wheat plants do in fact emit NH_3_ gas, genotypes from the high-GS group (Cadenza and Paragon) and low-GS group (Claire) were analysed using a Picarro real-time gas analyser. For this experiment, plants were grown under high-N and low-N conditions, with the latter treatment significantly reducing plant dry weight in all genotypes by about 50% (Supplementary Figure 5). Plants were supplied with the equivalent of 250 kg N ha^–1^ either as NH_4_NO_3_ or KNO_3_ in the morning, and gas emission was measured over a period of 24 h. Emission of NH_3_, as well as N_2_O, showed significant differences (*F*_181_ = 5.803, *p* < 0.05; *F*_150_ = 17.6975, *p* < 0.001, respectively) between plants and soil controls, with higher maximum emission from plants under high-N and low-N conditions (Figures 5A,B). Maximum NH_3_ gas emission occurred at about 5–6 h after N application, whereas maximum N_2_O emission occurred later, at about 15 h after N application (Figures 5C–E). Representative NH_3_ and N_2_O emission data generated from processed Picarro data (Paragon grown under low N conditions) are shown in Figures 5C,D. N_2_O emission was overall higher and within the ppm range, whereas NH_3_ gas emission was in the ppb range.

Regardless of N treatment, max NH_3_ emission was significantly different between plants grown under high-N and low-N conditions, with low-N plants showing higher NH_3_ emission (*F*_1.0289_ = 26.4939, *p* < 0.001; Figure 5F). Similarly, maximum N_2_O emission was significantly higher from plants grown under low-N conditions (*F*_0.7262_ = 32.8576, *p* < 0.001; Figure 5G). In the combined data of the three analysed genotypes, a significant effect of the N treatment was observed on N_2_O emission in plants grown under low-N conditions, with a significantly higher N_2_O emission after both, NH_4_NO_3_ and KNO_3_ application compared to water-treated control plants (Figure 5G). N_2_O emission was highest when KNO_3_ (*F*_0.7262_ = 6.0957, *p* < 0.05) was applied, in agreement with our current understanding that N_2_O is a by-product of nitrate reductase. In the combined data, there was no significant difference in NH_3_ emission between plants that received N and water control plants (Figure 5F). However, the log transformed NH_3_ emission data showed genotypic differences, with a significantly higher emission from the low GS-group representative variety Claire, compared to Cadenza and Paragon (Figure 5H). Likewise, N_2_O emission was significantly higher in Claire (Figure 5I). This was observed only in plants grown under low-N conditions but not in plants grown under high-N conditions (data not shown). To further investigate the effect of the N source on gas emission, Cadenza plants grown under low-N conditions were treated with urea and KNO_3_, with and without addition of the nitrate-reductase inhibitor Tungsten, respectively. In agreement with the expectation, the data show a significantly higher N_2_O when N was supplied as KNO_3_ compared with urea, and N_2_O emission was significantly reduced in the presence of Tungsten (Figure 5J).

**FIGURE 6:**
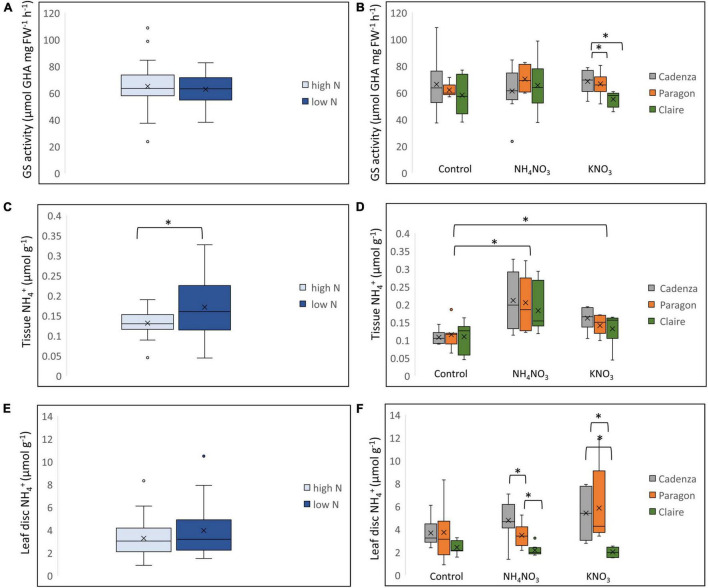
Differential N response in plants grown under high and low N conditions. Selected genotypes, Cadenza, Paragon and Claire, were grown under low-N and high-N conditions until the 3–4 tiller stage and then treated with the equivalent of 250 kg N ha^–1^ as NH_4_NO_3_ and KNO_3_, respectively. Water was used for control plants. GS activity **(A)**, leaf tissue NH_4_^+^ **(C)** and leaf-disc NH_4_^+^ **(E)** were measured and compared between low-N and high-N plants. The effect of different treatments on GS enzyme activity **(B)**, leaf tissue NH_4_^+^ **(D)** and leaf-disc NH_4_^+^ **(F)** was compared between the three genotypes. Asterisks indicate significant differences between means (*p* < 0.05) of each treatment.

To assess N-uptake and assimilation of the three genotypes under low-N and high-N growth conditions, leaf samples of the previous experiment were collected at the end of the gas measurement (at 24 h after N application) and analysed for GS activity, and tissue and leaf-disc NH_4_^+^ (Figure 6). The analysis of the combined data showed a significantly (*F*_0.59_ = 0.16, *p* < 0.01) higher tissue NH_4_^+^ content in plants grown under low-N conditions, though in the combined data there was no significant difference in GS activity or leaf-disc NH_4_^+^ (Figures 6A,C,E). However, when comparing the genotypes individually, GS activity in Claire was variable in response to NH_4_NO_3_ and was significantly lower compared to Cadenza and Paragon when N was supplied as KNO_3_ (Figure 6B). All genotypes showed a significant increase in tissue NH_4_^+^ after N application, and this was most pronounced when N was supplied as NH_4_NO_3_ (Figure 6D). Under the experimental conditions applied, there was no significant difference between the genotypes in leaf tissue NH_4_^+^ (Figure 6D), however, in contrast leaf-disc NH_4_^+^ was significantly lower in Claire compared to the other two genotypes, after both, NH_4_NO_3_ and KNO_3_ application (Figure 6F). This is in agreement with the data from the diversity study (Figure 4F) showing an overall lower leaf-disc NH_4_^+^ in the low-GS group.

An additional Picarro experiment was carried out to compare United Kingdom varieties with wheat from a different agro-ecological zone and for this, the Australian elite variety Mace (Wyalkatchem/Stylet), which has been shown to yield well across different Australian environments (SARDI Crop Harvest Report, 2015), was included in this study. It is noteworthy that compared to the United Kingdom, wheat systems in Australia are relatively low-N input and low-yielding, suggesting that N responses in Mace might be different compared to Cadenza and Paragon, which are adapted to high N levels. In agreement with that, the data show that at 24 h after N application, Mace had a significantly lower GS activity, as well as lower leaf-tissue and leaf-disc NH_4_^+^ content compared to the United Kingdom varieties (Supplementary Figures 6A–C). Likewise, NH_3_ and N_2_O gas emission were also significantly lower in Mace (Supplementary Figures 6D,E) suggesting that the N assimilation system in wheat is indeed plastic and subject to breeders’ selections.

## Discussion

The main objective of this study was to assess the immediate and short-term responses within wheat to N-fertiliser application at the vegetative stage and to see if there is variation within the N-assimilation capacity that can be used for improving N-fertiliser assimilation.

### Wheat Genotypes Differ in Their N-Fertiliser Response

An initial time course experiment was conducted to assess whether and how rapidly GS activity in wheat responds to N-fertiliser application and if genotypic differences could be observed. The data showed that the spring wheat variety Paragon was overall more responsive to N compared with Cadenza, i.e., GS activity increased as early as 6 h after N application and also responded to lower N doses. The subsequent extended study, including the two spring wheat varieties and eight winter wheat varieties, confirmed that genotypic differences do exist, though there was considerable variation within the data. However, in response to N application, two significantly distinct groups, a high-GS group and low-GS group, could be distinguished. Members of the high-GS group (including both, Cadenza and Paragon) showed a constitutively higher and more N-responsive GS activity and glutamine content in response to N compared to the low GS-group, but also an increased tissue NH_4_^+^ level. An increase in tissue NH_4_^+^ concentration when GS activity is high may seem counterintuitive, however, this has also been described in rice by Kumagai et al. (2011a) suggesting that plants store excess NH_4_^+^ in the leaf when N uptake exceeds the N-assimilation capacity, likely in the vacuoles (Howitt and Udvardi, 2000). The response to N-fertiliser application was markedly different in genotypes constituting the low-GS group, compared with the high-GS group, with the former showing negative regulation of GS activity, no or little increase in tissue NH_4_^+^ and low glutamine content. This, in conjunction with the observed increase in leaf-disc NH_4_^+^, suggests overall poor N assimilation capacity and potentially high N losses in this group.

A negative response of leaf GS activity to N-fertiliser application has previously been reported from durum wheat (Nigro et al., 2016) and bread wheat (Zhang M. et al., 2017). This was only observed in high-grain protein genotypes; however, the high-grain protein genotypes had an overall higher GS activity, similar to the constitutive differences between bread wheat genotypes reported here.

Recent detailed studies in wheat (Wei et al., 2020) showed an increase in GS2 protein in leaves in response to both, NH_4_^+^ and NO_3_^–^ application and this was confirmed by an in-gel GS activity assay showing increased GS2 activity in response to both N sources. In agreement with that, there was no significant differences between N sources in the genotypes analysed in the present study. Interestingly, Wei et al. showed that, in contrast to GS2, GS1 expression and activity showed differential N responses with a positive NH_4_^+^ response in roots and a positive NO_3_^+^ in shoots (Wei et al., 2020).

### Regulation of Glutamine Synthetase Is Complex, and Genes Are Highly Conserved

It has been shown that the response of GS activity to high leaf tissue NH_4_^+^ differs among plant species. Studies in lupin and mustard have found that high NH_4_^+^ tissue concentration was inhibitory (Ratajczak et al., 1981; Schmidt and Mohr, 1989), whilst it was stimulatory in sugar beet, mustard and pine (Vollbrecht et al., 1989; Mäck and Tischner, 1990). It was suggested that one of the underlying reasons for these conflicting reports may be related to the plant carbon (C) to N ratio, e.g., plants with low C:N ratios might lead to a deficiency in N-acceptor carbon skeletons. The complex interplay and potential limitation of C in N metabolism has been studied intensively in relation to the alanine aminotransferase (AlaAT) pathway in various crops (e.g., McAllister et al., 2012; Tiong et al., 2021).

In order to study the expression of the GS genes, we have extracted the sequences of the wheat GS1 and GS2 genes from available, but as yet unannotated, genome sequences from five wheat varieties, in addition to the available Chinese Spring reference genome. The models are therefore putative and will need to be experimentally validated. However, comparison of the predicted protein sequences revealed a near 100% sequence conservation between genotypes across the entire protein and there was no non-synonymous change that could explain differences in GS activities between the high-GS and low-GS activity group. GS enzymes are composed of eight monomers assembled into a complex, high-molecular holoenzyme and given its essential function, this high degree of conservation within wheat and across species is not surprising (Kumada et al., 1993; Bernard et al., 2008; Thomsen et al., 2014; Yang et al., 2016).

Analysis of GS gene expression showed an approximately six-fold higher expression of GS2 in leaves, which is in agreement with other studies in wheat (Bernard et al., 2008; Zhang M. et al., 2017; Wei et al., 2020) and rice (Zhao and Shi, 2006). Although GS2 expression was higher than GS1 overall in Cadenza and Paragon, which also had the highest GS activity, observed positive transcriptional changes after N application were largely not significant; suggesting that constitutive differences might be more relevant than N-triggered changes. Other studies report similar findings, with expression of GS genes being higher in an N-efficient compared to an N-inefficient genotype regardless of N treatment (Tian et al., 2015) and an overall higher expression was also observed in high grain-N genotypes compared with low grain-N genotypes (Nigro et al., 2016; Zhang M. et al., 2017).

There are very few studies in cereals that have assessed dynamic changes in GS gene expression that allow comparison with the short-term responses assessed here. The earliest analysis of N responses in wheat we could find was conducted at 10 days post fertiliser application showing an increase in GS transcript abundance (Nigro et al., 2019). Zhang Z. et al. (2017) monitored *TaGS1* and *TaGS2* transcript level in two wheat genotypes, grown under low- and high-N conditions, throughout development and in different tissues. The data showed a steep increase in leaf *GS2* expression at the jointing stage and this was higher under high N, whereas *GS1* expression was highest at 14 days post-anthesis, in agreement with their roles in N-assimilation and N-remobilisation, respectively. The detailed studies in wheat reported by Wei et al. (2020) were conducted with plants grown for 12 days under a range of different steady-stated N concentrations but nevertheless revealed interesting details on the differential regulation of GS1 and GS2 by different N sources, as mentioned above. Strikingly, the authors showed that GS2 is highly expressed in roots whilst they were unable to detect the corresponding protein, suggesting negative post-transcriptional regulation of TaGS2 in roots.

A study in rice assessed *GS1* and *GS2* gene expression at 6 h after N application to N-starved plants and the authors report an increase in the low-abundance *OsGln1;1* gene, however, in agreement with our data the highly expressed *OsGln1;2* gene, as well as *OsGln2* were not responsive to N application at 6 h (Zhao and Shi, 2006). In another study in rice, GS gene expression in roots in response N-fertiliser application was analysed, showing a significant increase in transcripts of *OsGln1;2*, which is the most abundant GS gene in roots, in response to NH_4_^+^ but not to NO_3_^–^. In contrast, expression of another *GS1* isoform (*OsGln1;1*) decreased in response to both N sources, whilst *GS2* remained unchanged (low expression in roots). The analysed GOGAT genes were all significantly suppressed (Zhao and Shi, 2006). In a time-course experiment in wheat conducted with plants grown at two different N levels revealed little differences in *GS1* and *GS2* expression though in flag leaves, *GS2* expression was higher under high N conditions (Wei et al., 2021b). In an RNA-seq study in durum wheat *GS1* and *GS2* expression in leaves did not respond to long-term N starvation, however, *GS1* expression in roots increased consistent with its role in N uptake (Curci et al., 2017). Taken together, data on the transcriptional regulation of GS genes in response to N are inconsistent, and other factors need to be considered, such as post-transcriptional regulation and homeolog-specific expression (Wei et al., 2020), as well as post-translational regulation (Finnemann and Schjoerring, 2000; Swarbreck et al., 2008).

In contrast to the inconclusive role of transcriptional regulation in response to N, our dry-down experiment showed a significant upregulation of *GS1* and downregulation of *GS2* demonstrating that *GS* genes, in principle, can be regulated at the transcriptional level. Downregulation of *GS2* was also observed in durum wheat exposed to osmotic stress, however, *GS1* expression and GS activity remained unchanged in the three analysed genotypes (Jallouli et al., 2019). In fact, high *GS* expression has been implicated with a beneficial role under drought stress in a recent study with simultaneous overexpression of *GS1;1* and *GS2* (James et al., 2018), and had previously been observed in naturally drought-tolerant rice (Singh and Ghosh, 2013). GS activity was also responsive to salinity stress and specifically increased in the oldest of three analysed leaves, possibly related to N remobilisation under stress (Carillo et al., 2008).

### N Fertiliser Application Leads to N-Gas Emission

Volatilisation of NH_3_ occurs when NH_4_^+^-generating processes exceed the re-assimilation capacity (Sutton et al., 1995) and was first described in relation to photorespiration in soybean (Weiland and Stutte, 1985) and spring wheat (Morgan and Parton, 1989). Since then it has been reported in barley (Mattsson et al., 1998), rice (Kumagai et al., 2011a,b), and many other species (see introduction), including Lolium and Bromus grass (Mattsson and Schjoerring, 2002). In the latter study, a significant correlation between high leaf tissue NH_4_^+^ and apoplastic NH_4_^+^ concentration was observed. From the apoplast, NH_4_^+^ can be emitted as NH_3_ gas via stomata (Husted and Schjoerring, 1995; Pearson et al., 1998; Mattsson and Schjoerring, 2002). Likewise, in rice high NH_3_ emission was observed at high tissue NH_4_^+^ concentrations and this was associated with genotypic differences observed between japonica rice and an aus-type landrace, which had low GS activity (Kumagai et al., 2011a).

These studies prompted us to assess if the observed differences in GS activity and leaf tissue NH_4_^+^ between the high GS-group and the low-GS group was associated with emission of NH_3_.

Due to the physico-chemical properties of NH_3_ gas it is difficult to measure emission directly from plants, and specialised equipment is required (Schjoerring et al., 1992). However, it has been suggested that tissue NH_4_^+^ concentration can be used to estimate the NH_3_ compensation point because it often increases proportionally to apoplastic NH_4_^+^ Mattsson and Schjoerring (2002) and Sutton et al. (2009) found that NH_3_ emission was closely correlated with NH_4_^+^ concentration in leaf tissue. Therefore, we used the leaf-disc assay where NH_4_^+^ is extracted from fresh, intact leaf-discs as a proxy for apoplastic NH_4_^+^ (see section “Materials and Methods” for details). In agreement with the above-mentioned studies, we found that leaf-disc NH_4_^+^ was significantly higher in the high-GS group, which also had a significantly higher leaf tissue NH_4_^+^. However, contrary to the expectation, there was no obvious increase in leaf-disc NH_4_^+^ after N application, although tissue NH_4_^+^ increased in this group. This suggests that genotypes in the high-GS group are indeed better able to assimilate and store excess NH_4_^+^ in contrast to the low-GS group. In the latter group, the N storage and assimilation capacity was possibly exceeded as indicated by lack of increase in leaf tissue NH_4_^+^ and low glutamine content. The increase in leaf-disc NH_4_^+^ might thus be a way to prevent accumulation of toxic cellular NH_4_^+^ levels.

Based on these indicative data, a follow-up study was conducted using a Picarro gas analyser to quantify NH_3_ as well as N_2_O gas emission. Cadenza and Paragon were selected as representatives of the high-GS group and Claire as a representative of the low-GS group.

Our current understanding of the roles of plants in N_2_O emission is still controversial with field-based studies suggesting that N_2_O is in fact produced by soil microorganisms and simply diffuses through plants (Chang et al., 1998; Rusch and Rennenberg, 1998; Baruah et al., 2010; Machacova et al., 2013; Bowatte et al., 2014; Wen et al., 2017). However, other studies using a range of different plant species and conducted under sterile conditions provided convincing evidence that the N_2_O gas indeed is derived from a plant-inherent process, and not from the soil microbiome or microbes present on leaves (Goshima et al., 1999; Hakata et al., 2003; Lenhart et al., 2019). Although the underlying mechanisms remain to be elucidated in detail, there is evidence that N_2_O can be produced from nitric oxide (NO) in the mitochondria of plants under hypoxic conditions (for a review see Timilsina et al., 2020) and this is also relevant in the context that NO acts as a second messenger in plants (for recent reviews see Astier et al., 2018; Gupta et al., 2020). However, more relevant in relation to this study is the finding that N_2_O emission was specifically measured only when NO_3_^–^ was applied as the N source, but not with NH_4_^+^ or glycine (Lenhart et al., 2019).

The data presented here are in support of the findings that N gas emission originates from plants since there was a significant difference between pots with plants and control pots, from which the above-ground plant tissue was removed immediately before the gas measurement. Since the roots were retained in the controls and similar microbiomes should be present in all pots, differences in gas emissions can be ascribed to activities from leaves. Overall, after N application, emission was higher in plants that had been grown under N-starvation conditions, suggesting that, within the duration of the experiments, the N-assimilation and -storage capacity in N-starved plants was insufficient, leading to higher N-gas emission. This is in agreement with the study from Schjoerring and Mattsson (2001) who reported higher NH_3_ losses from winter wheat that had received 75% of a standard N dose, compared to fully fertilised plants.

Likewise, differences between the genotypes included in this study were only observed in plants grown under N deficiency where NH_3_ and N_2_O emission was significantly higher in the low-GS genotype Claire compared to Paragon and Cadenza. However, analysis of the Australian variety Mace, which had low GS activity and also low gas emission, suggests that N gas emission is not simply a matter of GS activity.

Thus, more detailed comparative studies on N-gas emission in plants is clearly needed to establish the underlying mechanisms. However, the presented data are in support of studies by others that N-gas emission does occur from plants and that this is a response to N fertiliser application. Importantly, in this study we have found that emission of N_2_O is related to nitrate fertiliser application since it was not observed when N was applied as urea and was significantly reduced in the presence a nitrate reductase inhibitor, which is in agreement with the study by Lenhart et al. (2019) and our current understanding that N_2_O originates from plant NO_3_^–^ reduction. The role of N_2_O emission from plants in climate change has been realised (e.g., Smart and Bloom, 2001; Gogoi and Baruah, 2012; Lenhart et al., 2019) and needs to be addressed as a matter of urgency. The data presented here suggest that genotypic differences do exist that can be exploited for breeding and that N management and fertiliser choices would provide opportunities to reduce N_2_O emission from cropping systems.

## Conclusion

The data presented here are showing that natural variation of GS activity exists in wheat and that genotypes respond differently to N-fertiliser application. There is thus an opportunity to exploit this natural variation for breeding. However, for this it will be important to better understand the underlying mechanisms of post-transcriptional and post-translational regulation of GS enzyme activity. It will also be important to establish to what extend GS activity is directly relevant for preventing N-gas emission and if other factors, such as e.g., NH_4_^+^ and NO_3_^–^ storage capacity play important roles. This is not only relevant in relation to developing novel approaches towards enhancing crop N-fertiliser use efficiency, but even more so for the reduction of emission of the potent greenhouse gas N_2_O. Our data indicate that N_2_O emission is higher when N is applied to N-starved plants which warrants further investigations into the NO_3_^–^ uptake and assimilation pathways in plants, however, it also opens opportunities for addressing this by fertiliser management options and fertiliser choices.

## Data Availability Statement

The original contributions presented in the study are included in the article/Supplementary Material, further inquiries can be directed to the corresponding author.

## Author Contributions

SH was leading the project. MO conducted the glasshouse experiments. KH carried out the statistical analysis. AR conducted the field experiments. IC provided access to the Picarro instrument. DH and AT-B analysed the Picarro data. SH and MO wrote the manuscript. All authors contributed to the article and approved the submitted version.

## Conflict of Interest

The authors declare that the research was conducted in the absence of any commercial or financial relationships that could be construed as a potential conflict of interest.

## Publisher’s Note

All claims expressed in this article are solely those of the authors and do not necessarily represent those of their affiliated organizations, or those of the publisher, the editors and the reviewers. Any product that may be evaluated in this article, or claim that may be made by its manufacturer, is not guaranteed or endorsed by the publisher.

## Footnotes

1
https://plants.ensembl.org/Triticum_aestivum/Info/Index

2
https://grassroots.tools/

3
https://opendata.earlham.ac.uk/wheat/under_license/toronto/Clavijo_2017-04-25_Wheat-LoLa/

4
http://wheat-expression.com/

5
www.wheat-expression.com
