# Supplementary material for: Genetic Diversity in Nitrogen Fertiliser Responses and N Gas Emission in Modern Wheat
Source: Front Plant Sci. 2022 May 4;13:816475. doi: 10.3389/fpls.2022.816475 (PMC9137425; doi:10.3389/fpls.2022.816475)
Supplement: Supplementary file 1 [file Data_Sheet_1.docx]

***
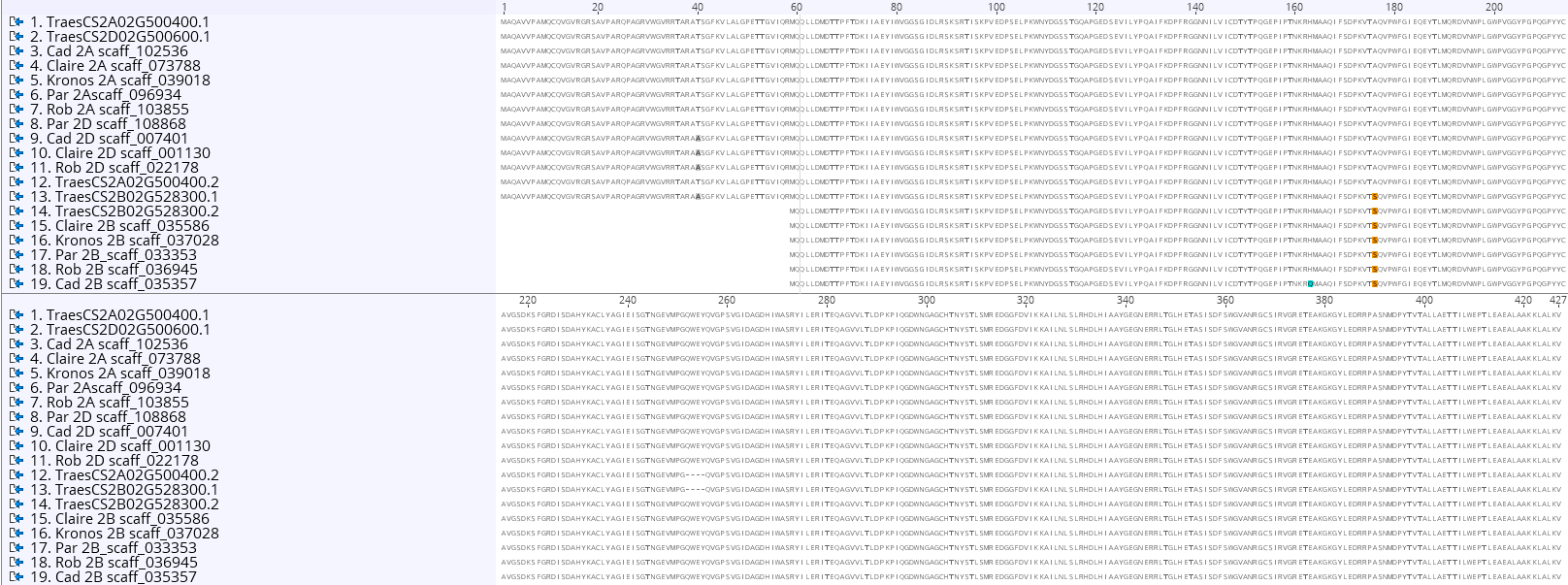
Supplementary material***

**Supplementary Figure 1. Alignment of the predicted protein sequences of *GS2* genes located on chromosome group 2.**

Putative *GS2* genomic sequences were identified in available genomic scaffolds of four bread wheat varieties (Cadenza, Paragon, Robigus, Claire) and the durum wheat Kronos (see Supplementary Table 1 and main text for details) Protein sequences were predicted using available gene models and sequence information from the wheat reference genome of Chinese Spring assessed via Ensembl Plants (<https://plants.ensembl.org/Triticum_aestivum/>). Sequence analysis and alignments were conducted using the Geneious software package (Geneious 10.2.6). Non-conserved amino acids are highlighted in different colours.

**
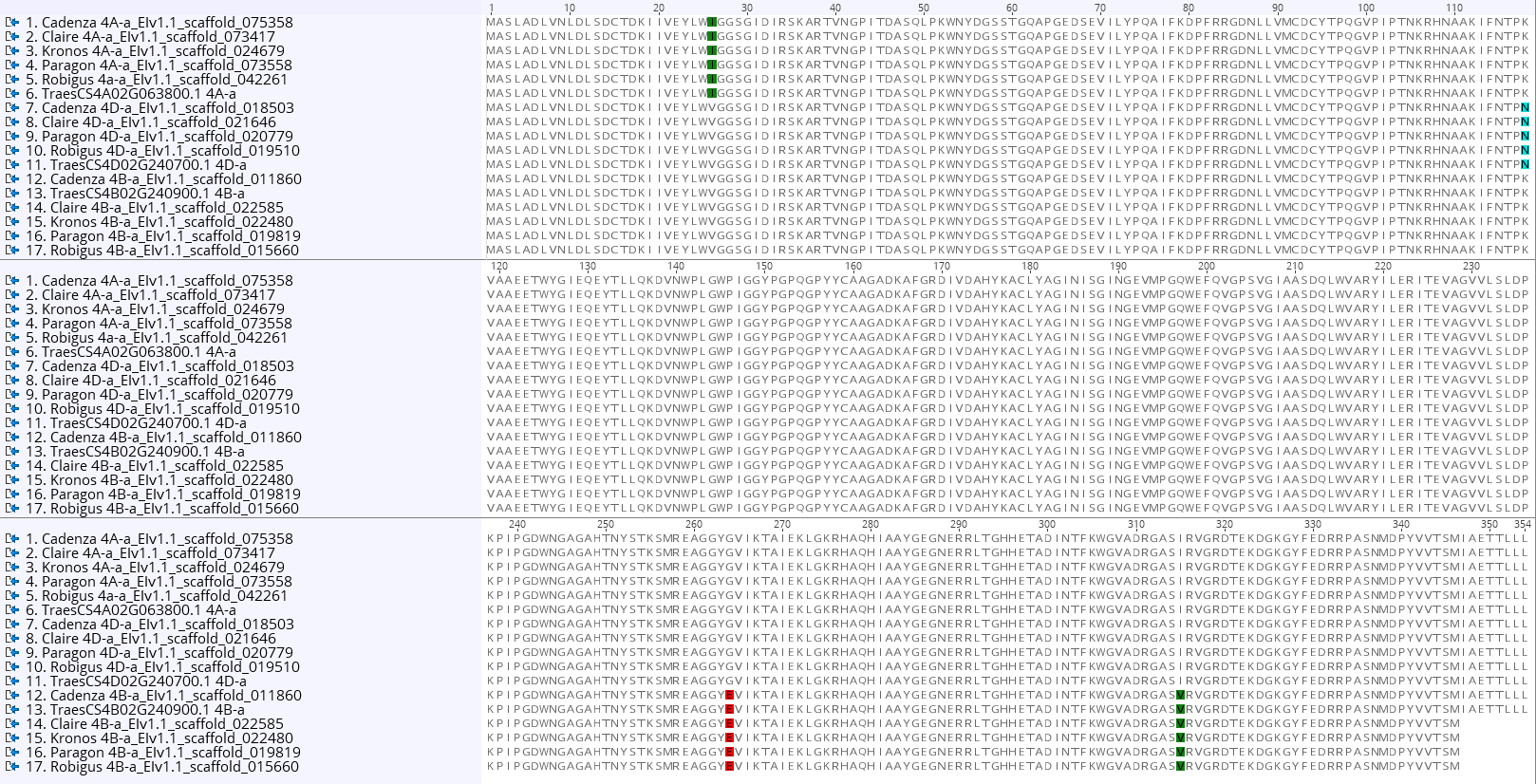
**

**Supplementary Figure 2a. Alignment of the predicted protein sequences of two *GS1;2* genes located on chromosome group 4**. See legend Figure S1.

**
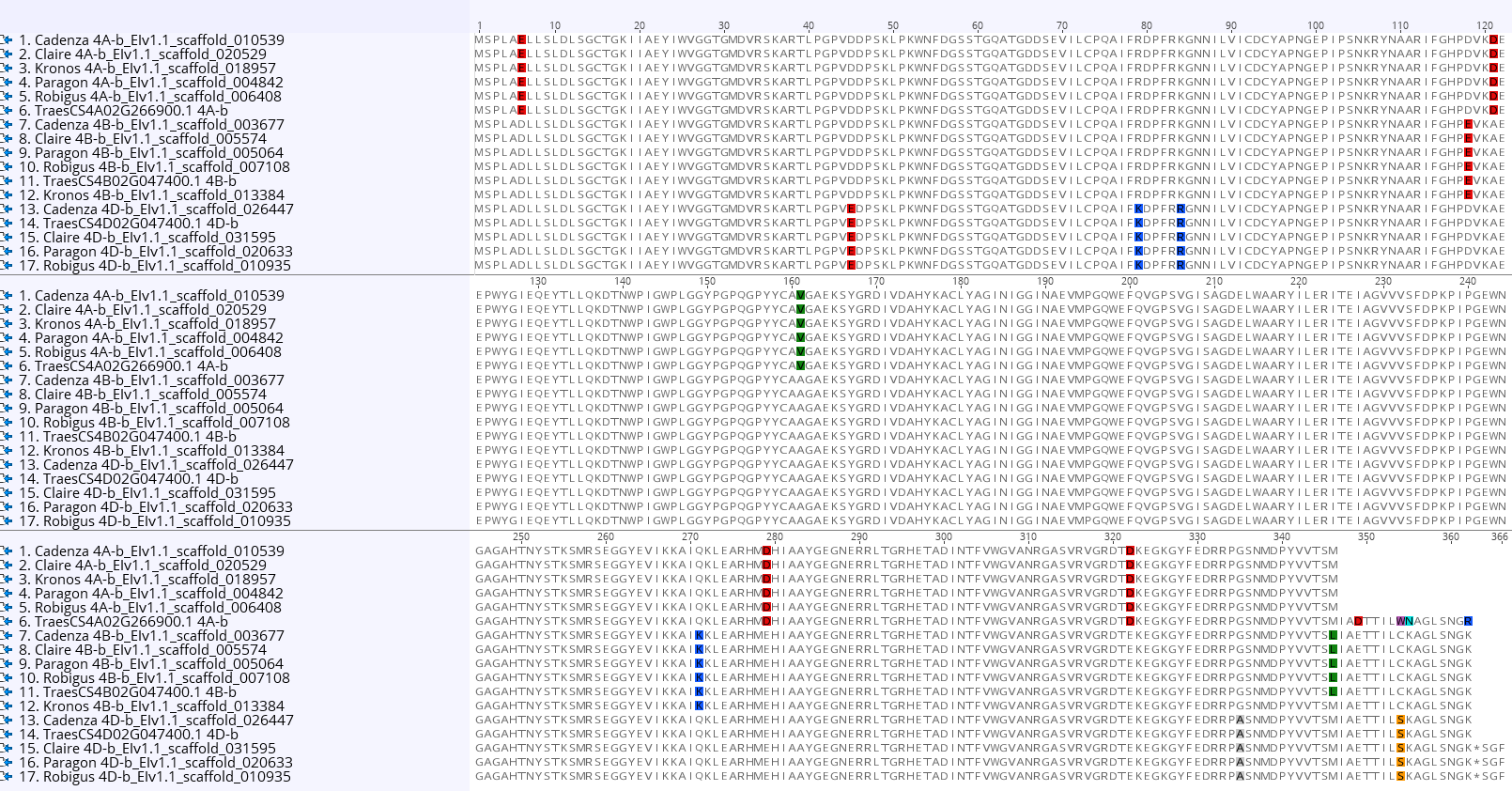
**

**Supplementary Figure 2b. Alignment of the predicted protein sequences of two *GS1;3* genes located on chromosome group 4.** See legend Figure S1.

**
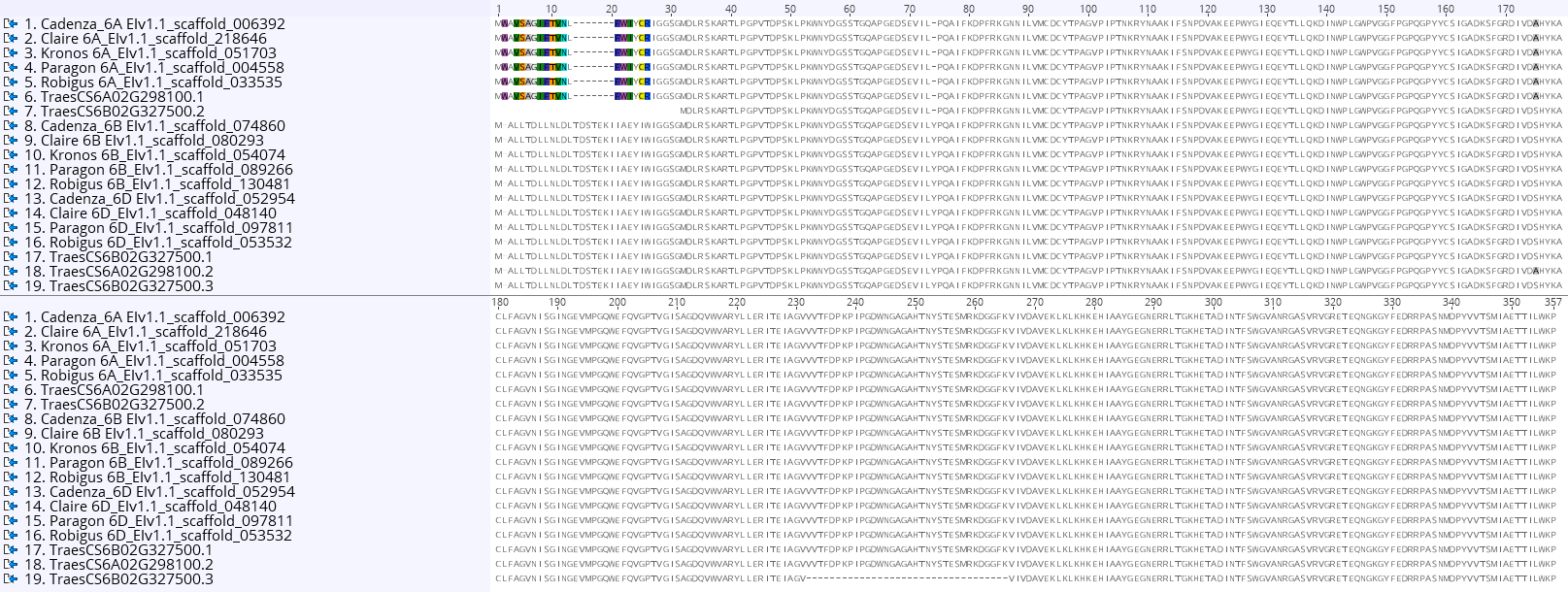
**

**Supplementary Figure 3. Alignment of the predicted protein sequences of *GS1;1* genes located on chromosome group 6.** See legend Figure S1.

**
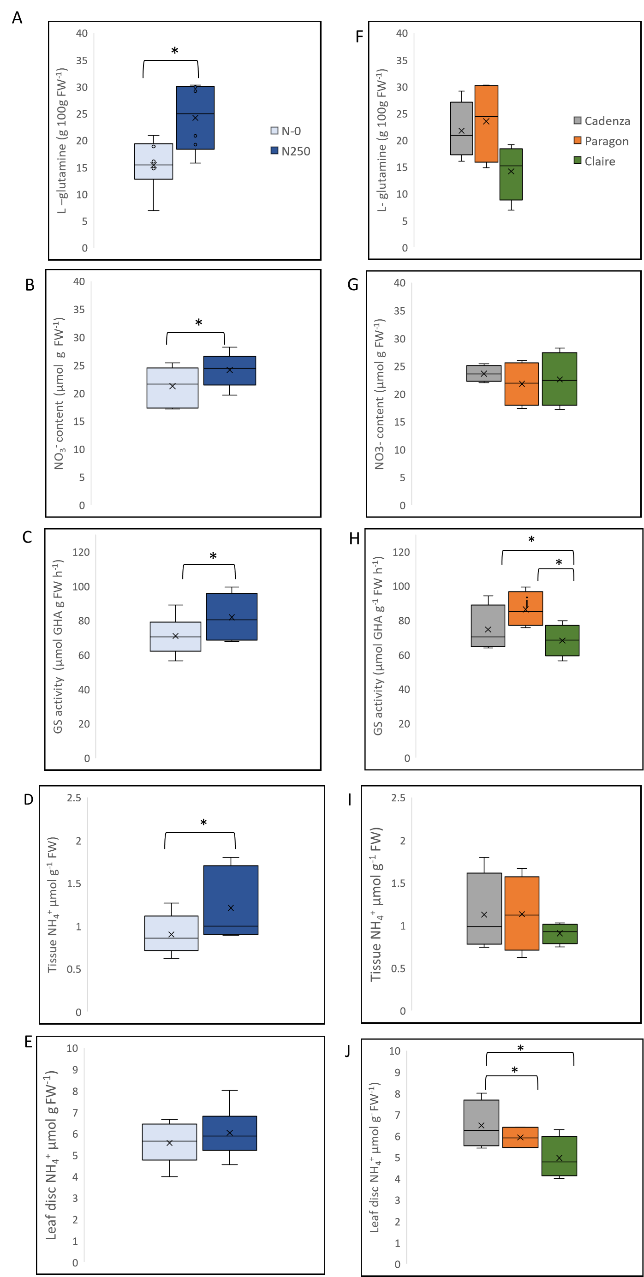
**

**Supplementary Figure 4. Glutamine, NO_3_^-^ and NH_4_^+^ content and GS activity in selected genotypes**.

Three wheat genotypes (Cadenza, Paragon, Claire) were grown under N-replete, fully fertilized conditions until the 2-3 tiller stage and then supplied with the equivalent of 250 kg N ha^-1^ as NH_4_NO_3_ (N250). Water was supplied to control plants (N-0) and always measured in parallel. Leaf samples were collected at 6h and 30h after the treatment to quantify the effect of the applied N on leaf L-glutamine (**A**) and NO_3_- (**B**) content, GS activity (**C**), and tissue (**D**) and leaf disc NH_4_^+^ (**E**) levels. The analysis of the corresponding genotypic differences is shown in (**F-J**) Each data represents the mean ± SE of four biological replicates. Asterisks indicate significant differences between means (p<0.05) of each treatment.


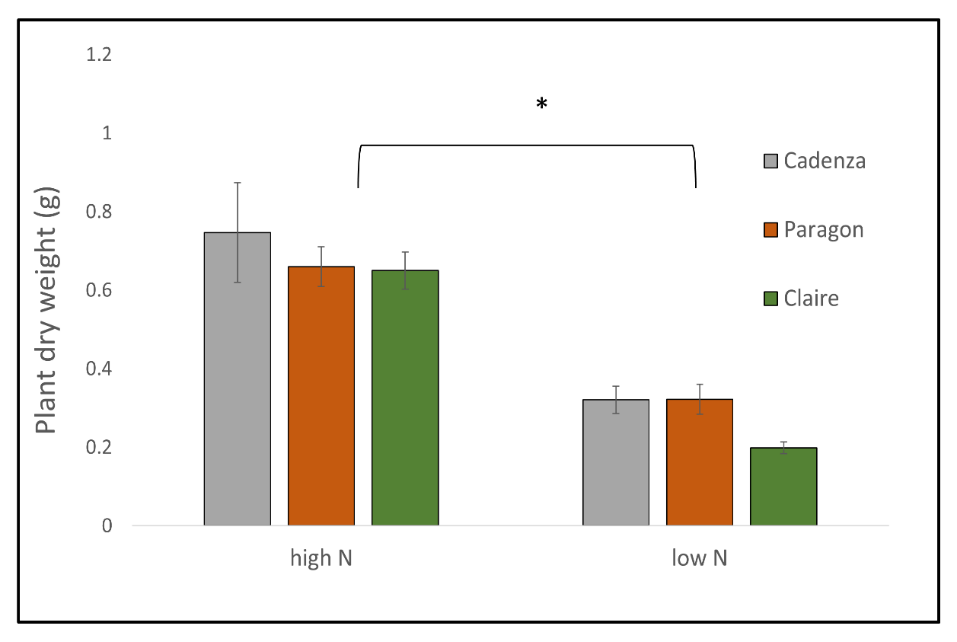


**Supplementary Figure 5. Effect of low-N growth conditions on plants dry weight**. At the end of the Picarro experiment (see material and methods in main text) plants were harvested and above-ground biomass was oven dried at 80°C for 24 hours and weighted. Each value represents the mean ± SE of six biological replicates. Asterisks indicate significant differences between means (p<0.05) of the two treatments.

**
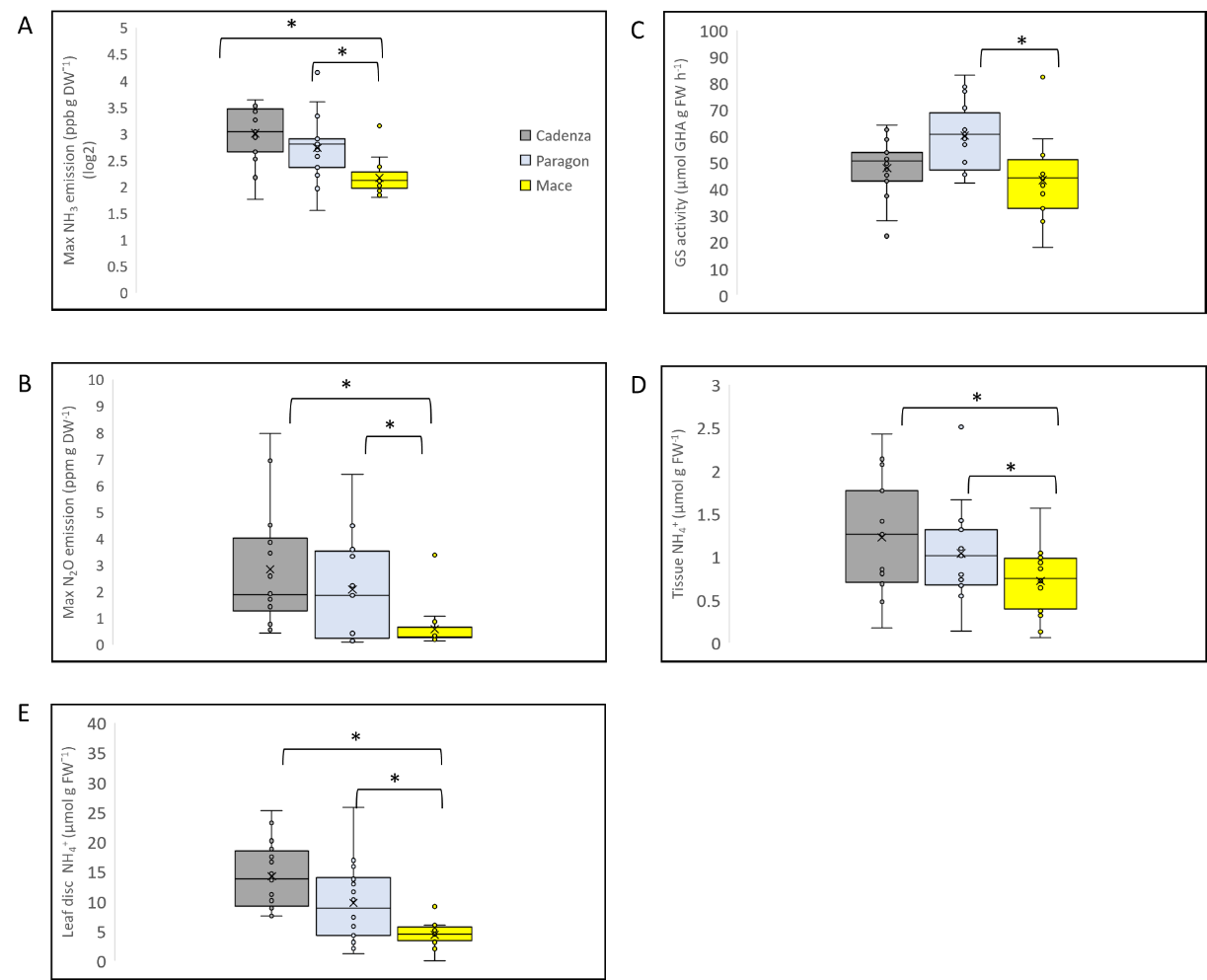
**

**Supplementary Figure 6. Comparison of N gas emission from UK and Australian wheat genotypes.**

The UK varieties Cadenza and Paragon and an Australian elite variety (Mace) were grown under N-replete, fully fertilized conditions. Plants at the 3-4 tiller stage were treated with the equivalent of 250 kg N ha^-1^ supplied as NH_4_NO_3_. Water was supplied to control plants. Picarro measurements were conducted as described in the main text to determine maximum NH_3_ (**D**) and N_2_O (**E**) emission. At the end of the experiment, leaf samples were taken to determine GS activity (**A**), leaf tissue NH_4_^+^ (**B**) and leaf disc NH_4_^+^ (**C**). There was no significant treatment effect in this experiment due to the high-N growth conditions and data were therefore combined to determine genotypic differences. Each value represents the mean ± SE of six biological replicates. Asterisks indicate significant differences between means (p<0.05) of the two treatments.

**Supplementary Table 1. Putative glutamine synthetase (GS) genes in different wheat genotypes**. GS1 and GS2 genomic sequences from the Chinese Spring reference genome (IWGSC, INSDC Assembly GCA_900519105.1; Jul 2018; https://plants.ensembl.org/Triticum_aestivum/Info/Index) were used as a query for the identification of scaffolds containing GS genes present in the sequenced durum wheat variety Kronos and four bread wheat varieties (Cadenza, Paragon, Robigus, Claire) available via grassroots (https://wheatis.tgac.ac.uk/grassroots-portal/blast; Bian. X, Tyrrell. S, Davey R.P. The Grassroots life science data infrastructure (2017). https://grassroots.tools). See material and methods in main text for details. Gene names in bold were adopted from Wei et al., (2021). Superseded gene names according to Bernard et al., (2008) are indicated in parenthesis below the respective gene accession numbers and were assigned based on a BLASTn search to identify genes with the highest sequence similarity.

| Chromosome |  |  | Scaffold |  |  |  |  | orientation | | GS gene located in scaffold based on Geneious alignment |
| --- | --- | --- | --- | --- | --- | --- | --- | --- | --- | --- |
|  |  |  | Triticum_aestivum_Cadenza_EIv1.1_scaffold_102536 | | | | | for |  | |
|  |  |  | Triticum_aestivum_Claire_EIv1.1_scaffold_073788 | | | | | for | *TaGS2-2A* | |
| Chr 2 group | **2A** |  | Triticum_aestivum_Paragon_EIv1.1_scaffold_096934 | | | | | rev | TraesCS2A02G500400 | |
|  |  |  | Triticum_aestivum_Robigus_EIv1.1_scaffold_103855 | | | | | for | (GS2a; DQ124212) | |
|  |  |  | Triticum_turgidum_Kronos_EIv1.1_scaffold_039018 | | | | | for | (GS2c; DQ124214; 99.7% 291 bp) | |
|  |  |  | Triticum_aestivum_Cadenza_EIv1.1_scaffold_035357 | | | | | for | |  |
|  |  |  | Triticum_aestivum_Claire_EIv1.1_scaffold_035586 | | | | | rev | | *TaGS2-2B* |
| Chr 2 group | **2B** |  | Triticum_aestivum_Paragon_EIv1.1_scaffold_033353 | | | | | rev | | TraesCS2B02G528300 |
|  |  |  | Triticum_aestivum_Robigus_EIv1.1_scaffold_036945 | | | | | for | | (GS2c; DQ124214 98.9%; 270bp) |
|  |  |  | Triticum_turgidum_Kronos_EIv1.1_scaffold_037028 | | | | | rev | |  |
|  |  |  | Triticum_aestivum_Cadenza_EIv1.1_scaffold_007401 | | | | | rev | | *TaGS2-2D* |
| Chr 2 group | **2D** |  | Triticum_aestivum_Claire_EIv1.1_scaffold_001130 | | | | | for | | TraesCS2D02G500600 |
|  |  |  | Triticum_aestivum_Paragon_EIv1.1_scaffold_108868 | | | | | rev | | (GS2b; DQ124213) |
|  |  |  | Triticum_aestivum_Robigus_EIv1.1_scaffold_022178 | | | | | for | |  |
|  |  |  | Triticum_aestivum_Cadenza_EIv1.1_scaffold_075358 | | | | | rev | |  |
|  |  |  | Triticum_aestivum_Claire_EIv1.1_scaffold_073417 | | | | | rev | | *TaGS1;2-4A* |
| Chr4 group1 | **4A** |  | Triticum_aestivum_Paragon_EIv1.1_scaffold_073558 | | | | | for | | TraesCS4A02G063800 |
|  |  |  | Triticum_aestivum_Robigus_EIv1.1_scaffold_042261 | | | | | rev | | none |
|  |  |  | Triticum_turgidum_Kronos_EIv1.1_scaffold_024679 | | | | | for | |  |
|  |  |  | Triticum_aestivum_Cadenza_EIv1.1_scaffold_011860 | | | | | for | |  |
|  |  |  | Triticum_aestivum_Claire_EIv1.1_scaffold_022585 | | | | | rev | | *TaGS1;2-4B* |
| Chr4 group1 | **4B** |  | Triticum_aestivum_Paragon_EIv1.1_scaffold_019819 | | | | | for | | TraesCS4B02G240900 |
|  |  |  | Triticum_aestivum_Robigus_EIv1.1_scaffold_015660 | | | | | rev | | (GSr1; AY491968) |
|  |  |  | Triticum_turgidum_Kronos_EIv1.1_scaffold_022480 | | | | | for | |  |
|  |  |  | Triticum_aestivum_Cadenza_EIv1.1_scaffold_018503 | | | | | rev | | *TaGS1;2-4D* |
| Chr4 group1 | **4D** |  | Triticum_aestivum_Claire_EIv1.1_scaffold_021646 | | | | | rev | | TraesCS4D02G240700 |
|  |  |  | Triticum_aestivum_Paragon_EIv1.1_scaffold_020779 | | | | | rev | | (GSr2; AY491969) |
|  |  |  | Triticum_aestivum_Robigus_EIv1.1_scaffold_019510 | | | | | rev | |  |
|  |  |  | Triticum_aestivum_Cadenza_EIv1.1_scaffold_010539 | | | | | for | |  |
|  |  |  | Triticum_aestivum_Claire_EIv1.1_scaffold_020529 | | | | | rev | | *TaGS1;3-4A* |
| Chr4 group2 | **4A** |  | Triticum_aestivum_Paragon_EIv1.1_scaffold_004842 | | | | | for | | TraesCS4A02G266900 |
|  |  |  | Triticum_aestivum_Robigus_EIv1.1_scaffold_006408 | | | | | for | | none |
|  |  |  | Triticum_turgidum_Kronos_EIv1.1_scaffold_018957 | | | | | for | |  |
|  |  |  | Triticum_aestivum_Cadenza_EIv1.1_scaffold_003677 | | | | | for | |  |
|  |  |  | Triticum_aestivum_Claire_EIv1.1_scaffold_005574 | | | | | rev | | *TaGS1;3-4B* |
| Chr4 group2 | **4B** |  | Triticum_aestivum_Paragon_EIv1.1_scaffold_005064 | | | | | rev | | TraesCS4B02G047400 |
|  |  |  | Triticum_aestivum_Robigus_EIv1.1_scaffold_007108 | | | | | rev | | (GSe2; AY491971) |
|  |  |  | Triticum_turgidum_Kronos_EIv1.1_scaffold_013384 | | | | | rev | |  |
|  |  |  | Triticum_aestivum_Cadenza_EIv1.1_scaffold_026447 | | | | | rev | | *TaGS1;3-4D* |
| Chr4 group2 | **4D** |  | Triticum_aestivum_Claire_EIv1.1_scaffold_031595 | | | | | for | | TraesCS4D02G047400 |
|  |  |  | Triticum_aestivum_Paragon_EIv1.1_scaffold_020633 | | | | | for | | (GSe1; AY491970) |
|  |  |  | Triticum_aestivum_Robigus_EIv1.1_scaffold_010935 | | | | | rev | |  |
|  |  |  | Triticum_aestivum_Cadenza_EIv1.1_scaffold_006392 | | | | | rev | |  |
|  |  |  | Triticum_aestivum_Claire_EIv1.1_scaffold_218646 | | | | | for | | *TaGS1;1-6A* |
| Chr 6 group | **6A** |  | Triticum_aestivum_Paragon_EIv1.1_scaffold_004558 | | | | | for | | TraesCS6A02G298100 |
|  |  |  | Triticum_aestivum_Robigus_EIv1.1_scaffold_033535 | | | | | rev | | (GS1a; DQ124209) |
|  |  |  | Triticum_turgidum_Kronos_EIv1.1_scaffold_051703 | | | | | for | |  |
|  |  |  | Triticum_aestivum_Cadenza_EIv1.1_scaffold_074860 | | | | | rev | |  |
|  |  |  | Triticum_aestivum_Claire_EIv1.1_scaffold_080293 | | | | | rev | | *TaGS1;1-6B* |
| Chr 6 group | **6B** |  | Triticum_aestivum_Paragon_EIv1.1_scaffold_089266 | | | | | rev | | TraesCS6B02G327500 |
|  |  |  | Triticum_aestivum_Robigus_EIv1.1_scaffold_130481 | | | | | for | | (GS1b; DQ124210) |
|  |  |  | Triticum_turgidum_Kronos_EIv1.1_scaffold_054074 | | | | | rev | |  |
|  |  |  | Triticum_aestivum_Cadenza_EIv1.1_scaffold_052954 | | | | | rev | | *TaGS1;1-6D* |
| Chr 6 group | **6D** |  | Triticum_aestivum_Claire_EIv1.1_scaffold_048140 | | | | | rev | | TraesCS6D02G383600LC |
|  |  |  | Triticum_aestivum_Paragon_EIv1.1_scaffold_097811 | | | | | for | | (GS1c; DQ124211) |
|  |  |  | Triticum_aestivum_Robigus_EIv1.1_scaffold_053532 | | | | | for | |  |

**Supplementary Table 2.** Data on N parameters across ten wheat genotypes

| **Genotype** | **Time point** | **Treatment** | **GS activity** (µmol GHA mg FW-1) | | **tissue NH4+** (µmol g-1) | |  | **Leaf disc NH4+** (µmol g-1) | |
| --- | --- | --- | --- | --- | --- | --- | --- | --- | --- |
|  |  |  | Data Mean ± Data SE | P-value (comparison to control) | Data Mean ± Data SE | P-value (comparison to control) |  | Data Mean ± Data SE | P-value (comparison to control) |
| Paragon | *6h* | *Water (control)* | 80.58 ± 6.59 |  | 1.27 ± 0.15 |  |  | 6.37 ± 0.71 |  |
| Paragon | *6h* | *NH4NO3* | 78.32 ± 5.03 | 0.8057 | 1.67 ± 0.18 | 0.3818 |  | 6.42 ± 0.67 | 0.9526 |
| Paragon | *6h* | *Urea* | 87.12 ± 10.78 | 0.4756 | 1.31 ± 0.18 | 0.9563 |  | 7.52 ± 1.24 | 0.7692 |
| Paragon | *30h* | *Water (control)* | 88.21 ± 9.07 |  | 0.62 ± 0.22 |  |  | 5.46 ± 0.71 |  |
| Paragon | *30h* | *NH4NO3* | 100.48 ± 8.44 | 0.1819 | 0.97 ± 0.16 | **0.0062** |  | 5.48 ± 0.67 | 0.9888 |
| Paragon | *30h* | *Urea* | 83.82 ± 8.47 | 0.6322 | 1.11 ± 0.21 | **0.0027** |  | 5.01 ± 1.24 | 0.6198 |
| Cadenza | *6h* | *Water (control)* | 61.33 ± 8.37 |  | 1.06 ± 0.1 |  |  | 6.67 ± 1.62 |  |
| Cadenza | *6h* | *NH4NO3* | 58.67 ± 7.59 | 0.7724 | 1.8 ± 0.36 | 0.1495 |  | 8.02 ± 0.89 | 0.3319 |
| Cadenza | *6h* | *Urea* | 69.54 ± 6.68 | 0.3707 | 0.69 ± 0.11 | 0.1293 |  | 6.89 ± 0.91 | 0.7518 |
| Cadenza | *30h* | *Water (control)* | 82.09 ± 9.33 |  | 0.74 ± 0.16 |  |  | 5.84 ± 1.46 |  |
| Cadenza | *30h* | *NH4NO3* | 91.07 ± 7.62 | 0.3280 | 0.91 ± 0.21 | 0.9521 |  | 5.44 ± 1.14 | 0.8909 |
| Cadenza | *30h* | *Urea* | 82.51 ± 8.04 | 0.9641 | 1.01 ± 0.12 | 0.2526 |  | 6.9 ± 1.42 | 0.4555 |
| Soissons | *6h* | *Water (control)* | 72.54 ± 8.04 |  | 0.63 ± 0.08 |  |  | 3.57 ± 0.59 |  |
| Soissons | *6h* | *NH4NO3* | 70.42 ± 3.79 | 0.8173 | 0.53 ± 0.07 | 0.5552 |  | 4.31 ± 1.08 | 0.7240 |
| Soissons | *6h* | *Urea* | 72.06 ± 6.65 | 0.9580 | 0.62 ± 0.13 | 0.7520 |  | 5.51 ± 0.66 | 0.1607 |
| Soissons | *30h* | *Water (control)* | 93.98 ± 2.71 |  | 2.67 ± 0.69 |  |  | 10.01 ± 0.84 |  |
| Soissons | *30h* | *NH4NO3* | 85.94 ± 7.67 | 0.3811 | 6.45 ± 0.56 | **0.0021** |  | 2.85 ± 1.3 | **0.0000** |
| Soissons | *30h* | *Urea* | 83.74 ± 4.49 | 0.2647 | 9.07 ± 1.02 | **0.0001** |  | 8.03 ± 0.9 | 0.3142 |
| XL-19 | *6h* | *Water (control)* | 61.73 ± 9.84 |  | 0.33 ± 0.09 |  |  | 7.95 ± 1.46 |  |
| XL-19 | *6h* | *NH4NO3* | 62.61 ± 4.47 | 0.9239 | 0.58 ± 0.1 | **0.0160** |  | 6.02 ± 0.92 | 0.3004 |
| XL-19 | *6h* | *Urea* | 81.81 ± 6.16 | **0.0293** | 0.55 ± 0.08 | **0.0190** |  | 5.23 ± 1.2 | 0.0961 |
| XL-19 | *30h* | *Water (control)* | 88.83 ± 6.92 |  | 0.92 ± 0.08 |  |  | 4.01 ± 0.36 |  |
| XL-19 | *30h* | *NH4NO3* | 83 ± 1.73 | 0.5969 | 1.21 ± 0.16 | 0.4181 |  | 4.65 ± 0.62 | 0.6758 |
| XL-19 | *30h* | *Urea* | 75.22 ± 3.93 | 0.1472 | 0.92 ± 0.1 | 0.9875 |  | 3.41 ± 0.89 | 0.4708 |
| Robigus | *6h* | *Water (control)* | 60.65 ± 7.86 |  | 0.81 ± 0.04 |  |  | 4.45 ± 0.84 |  |
| Robigus | *6h* | *NH4NO3* | 82.26 ± 6.85 | **0.0190** | 1.56 ± 0.28 | 0.1084 |  | 3.22 ± 0.64 | 0.3812 |
| Robigus | *6h* | *Urea* | 66.11 ± 3.74 | 0.5519 | 1.1 ± 0.34 | 0.7640 |  | 4.09 ± 0.66 | 0.8646 |
| Robigus | *30h* | *Water (control)* | 78.27 ± 7.38 |  | 1.05 ± 0.22 |  |  | 8.92 ± 0.59 |  |
| Robigus | *30h* | *NH4NO3* | 92.41 ± 7.22 | 0.1240 | 1.59 ± 0.05 | 0.0927 |  | 10.75 ± 0.7 | 0.3863 |
| Robigus | *30h* | *Urea* | 66.89 ± 7.87 | 0.2157 | 4.72 ± 1.43 | **0.0000** |  | 9.18 ± 0.32 | 0.8813 |
| Rialto | *6h* | *Water (control)* | 48.71 ± 2.85 |  | 1.47 ± 0.11 |  |  | 5.18 ± 1.39 |  |
| Rialto | *6h* | *NH4NO3* | 63.82 ± 5.22 | 0.1004 | 1.6 ± 0.22 | 0.8717 |  | 4.78 ± 0.88 | 0.9254 |
| Rialto | *6h* | *Urea* | 74.86 ± 7.07 | **0.0046** | 1.61 ± 0.3 | 0.9039 |  | 5.35 ± 1.31 | 0.8839 |
| Rialto | *30h* | *Water (control)* | 70.78 ± 7.6 |  | 1.03 ± 0.09 |  |  | 5.79 ± 1.01 |  |
| Rialto | *30h* | *NH4NO3* | 72.03 ± 6.46 | 0.8915 | 2.39 ± 1.24 | 0.1799 |  | 5.1 ± 1.05 | 0.6026 |
| Rialto | *30h* | *Urea* | 70.76 ± 4.39 | 0.9986 | 4.3 ± 1.41 | **0.0005** |  | 4.81 ± 0.56 | 0.5777 |
| Claire | *6h* | *Water (control)* | 62.73 ± 4.19 |  | 0.75 ± 0.07 |  |  | 4 ± 2.41 |  |
| Claire | *6h* | *NH4NO3* | 58.85 ± 7.84 | 0.6725 | 1.03 ± 0.14 | 0.3982 |  | 6.3 ± 0.74 | **0.0196** |
| Claire | *6h* | *Urea* | 75.1 ± 5.33 | 0.1782 | 1.2 ± 0.09 | 0.1392 |  | 5.52 ± 1.39 | 0.0874 |
| Claire | *30h* | *Water (control)* | 76 ± 5.52 |  | 0.96 ± 0.11 |  |  | 5.02 ± 0.74 |  |
| Claire | *30h* | *NH4NO3* | 72.22 ± 5.89 | 0.6804 | 0.89 ± 0.08 | 0.8352 |  | 4.56 ± 0.69 | 0.7392 |
| Claire | *30h* | *Urea* | 64.58 ± 6.22 | 0.2360 | 1.06 ± 0.08 | 0.7241 |  | 4.14 ± 0.52 | 0.5658 |
| Hereward | *6h* | *Water (control)* | 64.5 ± 2.75 |  | 0.76 ± 0.1 |  |  | 4.69 ± 1.39 |  |
| Hereward | *6h* | *NH4NO3* | 63.4 ± 3.46 | 0.9446 | 1.12 ± 0.14 | 0.2450 |  | 7.06 ± 1.37 | 0.0746 |
| Hereward | *6h* | *Urea* | 66.08 ± 4.31 | 0.8412 | 1.08 ± 0.1 | 0.2874 |  | 2.21 ± 0.39 | 0.1106 |
| Hereward | *30h* | *Water (control)* | 74.37 ± 8.91 |  | 0.98 ± 0.14 |  |  | 4.46 ± 0.78 |  |
| Hereward | *30h* | *NH4NO3* | 57 ± 7.36 | **0.0590** | 1.32 ± 0.28 | 0.9782 |  | 6.57 ± 1.4 | 0.5680 |
| Hereward | *30h* | *Urea* | 60.52 ± 4.82 | 0.1317 | 1.14 ± 0.1 | 0.5558 |  | 4.61 ± 1.09 | 0.9983 |
| Alchemy | *6h* | *Water (control)* | 53.04 ± 5.08 |  | 0.64 ± 0.11 |  |  | 1.94 ± 0.51 |  |
| Alchemy | *6h* | *NH4NO3* | 53.73 ± 3.7 | 0.9397 | 0.76 ± 0.16 | 0.8566 |  | 5.82 ± 0.82 | **0.0023** |
| Alchemy | *6h* | *Urea* | 64.4 ± 2.08 | 0.2383 | 0.61 ± 0.16 | 0.3707 |  | 5.22 ± 2.86 | **0.0557** |
| Alchemy | *30h* | *Water (control)* | 77.81 ± 8.4 |  | 0.84 ± 0.18 |  |  | 4.46 ± 0.32 |  |
| Alchemy | *30h* | *NH4NO3* | 70.19 ± 5.37 | 0.4064 | 0.58 ± 0.06 | 0.3575 |  | 3.42 ± 0.39 | 0.4185 |
| Alchemy | *30h* | *Urea* | 61.89 ± 5.99 | 0.0835 | 0.5 ± 0.18 | **0.0084** |  | 9.21 ± 1.27 | **0.0085** |
| Brompton | *6h* | *Water (control)* | 63.33 ± 7.32 |  | 1.12 ± 0.2 |  |  | 3.52 ± 1.11 |  |
| Brompton | *6h* | *NH4NO3* | 54.75 ± 3.28 | 0.3502 | 1.21 ± 0.17 | 0.7171 |  | 3.88 ± 0.8 | 0.6927 |
| Brompton | *6h* | *Urea* | 58.02 ± 6.59 | 0.5631 | 1.19 ± 0.15 | 0.7272 |  | 3.23 ± 0.45 | 0.9881 |
| Brompton | *30h* | *Water (control)* | 62.62 ± 7.44 |  | 5.2 ± 1.44 |  |  | 6.61 ± 0.45 |  |
| Brompton | *30h* | *NH4NO3* | 61.77 ± 1.41 | 0.9261 | 1.99 ± 0.13 | **0.0240** |  | 6.92 ± 0.91 | 0.8976 |
| Brompton | *30h* | *Urea* | 55.11 ± 5.72 | 0.4129 | 0.5 ± 0.12 | **0.0000** |  | 6.26 ± 0.87 | 0.7955 |
